# The cell surface marker CD36 selectively identifies matured, mitochondria-rich hPSC-cardiomyocytes

**DOI:** 10.1038/s41422-020-0292-y

**Published:** 2020-03-10

**Authors:** Ellen Ngar-Yun Poon, Xiao-ling Luo, Sarah E. Webb, Bin Yan, Rui Zhao, Stanley Chun Ming Wu, Yong Yang, Peng Zhang, Huajun Bai, Jiaofang Shao, Ching Man Chan, Godfrey Chi-Fung Chan, Suk Ying Tsang, Rebekah L. Gundry, Huang-Tian Yang, Kenneth R. Boheler

**Affiliations:** 1 Department of Medicine and Therapeutics, and Centre for Cardiovascular Genomics and Medicine, The Chinese University of Hong Kong (CUHK), HKSAR, China; 2 CAS Key Laboratory of Tissue Microenvironment and Tumor, Laboratory of Molecular Cardiology, Shanghai Institute of Nutrition and Health, University of Chinese Academy of Sciences (CAS), CAS, Shanghai, China; 3 Division of Life Science and State Key Laboratory of Molecular Neuroscience, The Hong Kong University of Science and Technology, HKSAR, China; 4 Department of Computer Science, The University of Hong Kong (HKU), HKSAR, China; 5 Intervention and Cell Therapy Center, Peking University Shenzhen Hospital, Shenzhen, China; 6 School of Life Sciences, CUHK, HKSAR, China; 7 Department of Paediatrics and Adolescent Medicine, HKU, HKSAR, China; 8 School of Biomedical Sciences, HKU, HKSAR, China; 9 SUSTech Academy for Advanced Interdisciplinary Studies, Southern University of Science and Technology (SUSTech), Shenzhen, China; 10 Department of Bioinformatics, School of Biomedical Engineering and Informatics, Nanjing Medical University, Nanjing, Jiangsu China; 11 State Key Laboratory of Agrobiotechnology, CUHK, HKSAR, China; 12 Department of Biochemistry and Center for Biomedical Mass Spectrometry Research, Medical College of Wisconsin, Milwaukee, WI USA; 13 Institute for Stem Cell and Regeneration Medicine, CAS, Beijing, China; 14 Division of Cardiology, Department of Medicine, and Department of Biomedical Engineering, The Johns Hopkins University, Baltimore, MD USA; 15 Present Address: CardiOmics Program, Center for Heart and Vascular Research; Division of Cardiovascular Medicine; and Department of Cellular and Integrative Physiology, University of Nebraska Medical Center, Omaha, NE USA

**Keywords:** Stem cells, Stem-cell differentiation

Dear Editor,

Human pluripotent stem cell (hPSC)-derived cardiomyocytes (CMs) are of significant translational value to in vitro studies of human cardiac development, drug and cardiotoxicity testing and cardiac disease modelling. Differentiation of hPSCs to CMs, however, yields mixed cultures of atrial-, ventricular-, and pacemaker-like cells as well as non-CMs in variable proportions.^1^ Strategies to enrich CMs from non-CMs and to generate ventricular versus atrial cells have been successful; however, enriched hPSC-CMs are developmentally immature and fail to recapitulate key functional traits that are fundamental to the (patho)physiology of adult CMs.^1,2^ Moreover, these strategies do not adequately address experimental variabilities caused by differences in the genomes and differentiation capabilities of diverse hPSC lines. One validated approach that overcomes issues of cell heterogeneity and experimental variability is immunophenotyping; however, accessible markers suitable for defining mature, live CMs are lacking. Although cell surface markers such as SIRPA(CD172a) and VCAM1(CD106)^3,4^ have been used to sort for hPSC-CMs, they do not distinguish between maturation states. Here, we report the identification of CD36 as a cell surface marker of maturation, which can be used to reduce experimental variability and improve drug screens.

To identify cell surface proteins informative of more mature CMs, we qualitatively profiled the surfaceome of cardiac Troponin T (TNNT2)-positive (>95%) human embryonic stem cell (hESC) (H7)-derived CMs (Supplementary information, Fig. S1) using mass spectrometry-based cell surface capture (CSC) technology.^5^ We identified 525 N-glycoproteins composed of membrane (85.5%), glycosylphosphatidylinositol-anchored (5.9%), and extracellular matrix (ECM) (5.0%) proteins. Cluster of differentiation (CD) molecules comprised 14% of the total glycoproteins identified (Supplementary information, Table S1). Comparisons with the Cell Surface Protein Atlas (CSPA) revealed 12 CD molecules with cardiac-prevalent expression (Supplementary information, Fig. S2).^6^ Among these, only CD36, a fatty acid translocase implicated in the uptake of long-chain fatty acids (FAs) in adult heart,^7^ was up-regulated in CMs as a function of differentiation time and differentially present on the surface of sub-populations of CMs (Fig. 1a, b, Supplementary information, Fig. S3). CD36 is present on the surface of multiple cell types including CMs, hematopoietic cells and adipocytes (Supplementary information, Fig. S2), but it is absent from undifferentiated hPSCs (Fig. 1b) and fibroblasts (not shown). Comparisons with public datasets show that CD36 transcripts increase in mouse and human hearts during in vivo development and are more abundant in ventricles than in atria from late embryonic mouse hearts (Supplementary information, Fig. S4).

**Fig. 1:**
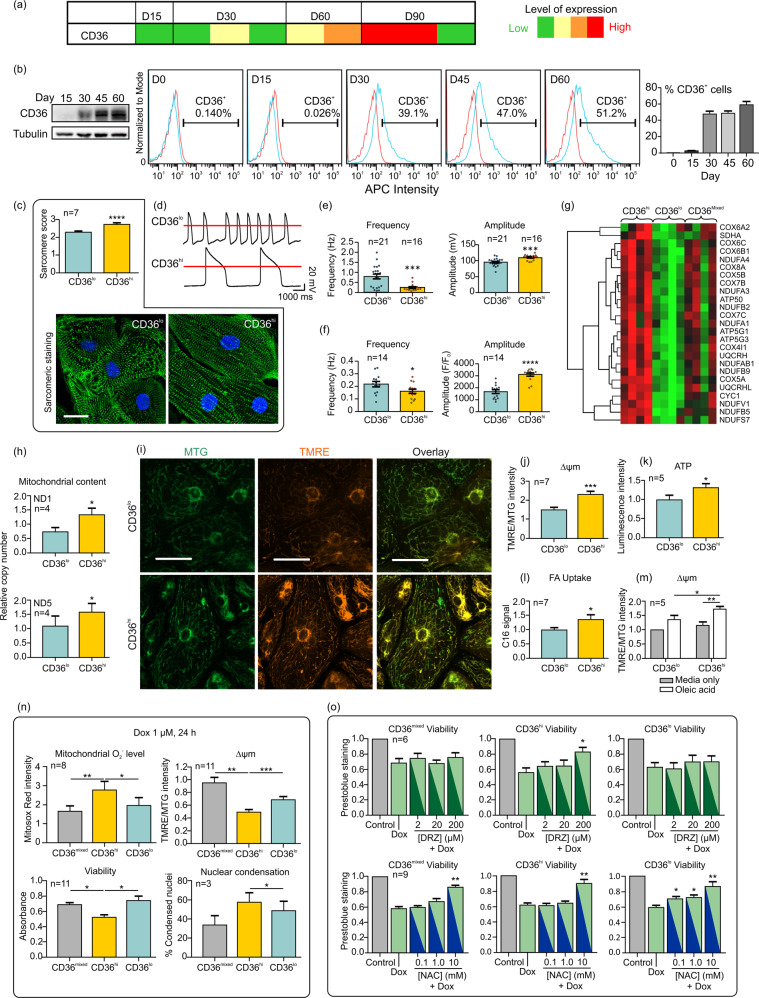
CD36 defines sub-populations of CMs with unique phenotypes that co-exist in culture. **a** Heat map showing the normalized CD36 peptide counts identified by mass spectrometry as a function of differentiation time. **b** Western blot and flow cytometry analyses showing increased levels and surface expression of CD36, respectively. Isotype control and CD36-APC staining are shown in red and blue. Summary graph quantitatively showing the proportion of CD36^+^CMs with time in culture. **c** Anti-α-actinin antibody (green) and DAPI (blue) staining reveals better sarcomeric structures and scores in CD36^hi^ than CD36^lo^ CMs. Scale bar = 20 μm. **d** Action potential (AP) tracings of CD36 subpopulations and **e** AP frequencies and amplitudes in CD36^lo^ and CD36^hi^ CMs. **f** Ca^2+^ transient frequencies and amplitudes in CD36^lo^ and CD36^hi^ CMs. **g** Gene ontology analyses of RNA-seq data show differential expression of mitochondrial gene transcripts among the 3 sub-populations. **h** Mitochondrial DNA content expressed as a function of mitochondrial gene (ND1 and ND5) copy numbers normalized to GAPDH and relative to unsorted CMs. **i** Images of cells stained with MTG (green) and TMRE (red) dyes, labelling mitochondria and polarized mitochondria, respectively. Scale bar = 50 μm. **j** Averaged Δψm data calculated as TMRE/MTG. **k** Averaged ATP production measured by luminescence. **l** Uptake of the palmitic acid analog, expressed as fluorescence, in CD36^lo^ and CD36^hi^ CMs. **m** The effects of fuel utilization on Δψm were measured ± oleic acid supplementation and normalized to CD36^lo^ CMs. Increased Δψm after oleic acid supplementation indicates elevated fatty acid utilization. **n** Effect of doxorubicin on CD36^mixed^, CD36^hi^ and CD36^lo^ CMs. Mitochondrial O_2_^−^, Δψm, viability and nuclear condensation were measured using mitosox red, TMRE/MTG, XTT and Hoechst, respectively. Data are normalized to untreated cells. **o** CD36^mixed^, CD36^hi^ and CD36^lo^ CMs were pre-treated with dexrazoxane (DRZ) and N-acetylcysteine (NAC) for one hour prior to co-treatment with doxorubicin (1 µM, 24 h). *P < 0.05, **P < 0.01, ***P < 0.001, ****P < 0.0001.

Human PSC-CMs could be sorted into CD36^hi^ or CD36^lo^ cell populations with unique traits. Differentiation day (D) 45 CD36^hi^ cells were enriched for α-actinin^+^ CMs, whereas CD36^lo^ cells contained both α-actinin^+^ CMs and α-actinin^−^ non-CMs (Supplementary information, Fig. S5). Sorting, after co-staining of CD172a (Supplementary information, Fig. S6) or pre-treatment with lactate, could eliminate most non-CMs. The percentages of CMs positive for CD36 at D45 ± 5 ranged from 34–78, 18–72, 30–81%, to 42–68% in differentiated cultures of H7 and H9 ESC lines and MD1- and JHU001-induced pluripotent stem cell (iPSC) lines, respectively (Supplementary information, Fig. S7). CMs derived from hPSC lines thus display a large degree of intra- and inter-line variability with respect to CD36. In atrial-like CMs induced with retinoic acid (RA), the fluorescent signal intensities for CD36^hi^ CMs were lower than those observed from ventricular CMs derived from H7 or JHU001 lines (Supplementary information, Fig. S8).

Sorted CD36^hi^ CMs (Supplementary information, Fig. S6 for gating) have traits characteristic of a more mature state. CD36^hi^ CMs have similar morphology but on average a 10% greater mean surface area, compared to CD36^lo^ CMs (Supplementary information, Fig. S9a, b). At D45, little to no cell proliferation could be demonstrated, and higher percentages of binucleate cells negative for Ki67 were observed in the CD36^hi^ (9.9 ± 0.8) *versus* CD36^lo^ (4.5 ± 0.8) CMs (Supplementary information, Fig. S9c–e). This latter trait correlated with elevated centromeric/kinetochore RNAs for CENPH and CENPM in CD36^hi^ CMs (Supplementary information, Tables S2 and S3). Structurally, CD36^hi^ CMs have more organized sarcomeres with elevated sarcomeric scores (Fig. 1c) relative to CD36^lo^ CMs, but myofibril widths did not significantly differ (CD36^hi^: 1.17 ± 0.23, *n* = 14; CD36^lo^: 1.28 ± 0.21, *n* = 16). Organized sarcomeres with H zones, A bands, and Z-disks could be clearly discerned in CD36^hi^ CMs (Supplementary information, Fig. S9f). Early ventricular hPSC-CMs express both the ventricular and atrial myosin regulatory light chain isoforms (MLC2V and MLC2A), but in more mature ventricular CMs, the cells express predominantly MLC2V. Consistently, the MLC2V to MLC2A ratio was higher in CD36^hi^ than in CD36^lo^ CMs (Supplementary information, Fig. S9g). Transcripts encoded from developmentally regulated genes such as cardiac troponin I and MLC2V were also increased by 1.40 ± 0.14 and 6.80 ± 3.56 fold, respectively in CD36^hi^ *versus* CD36^lo^ CMs (Supplementary information, Fig. S9h). T-tubules were not observed in any cell subpopulation.

Functionally, CD36^hi^ CMs (±RA) from all hPSC lines tested had significantly lower spontaneous beating frequencies (Supplementary information, Fig. S10a) and a decreased incidence of beating rate variability (i.e., arrhythmias) relative to CD36^lo^ CMs. Electrophysiologically, all cells differentiated without RA, displayed ventricular-like action potentials (AP). CD36^hi^ CMs had a prolonged plateau phase, a decreased diastolic depolarization (DD) slope and similar maximum diastolic potential (MDP) compared with CD36^lo^ CMs (Fig. 1d, Supplementary information, S10b). Relative to CD36^lo^ CMs, CD36^hi^ CMs had significantly increased AP amplitudes, reduced spontaneous firing frequencies, longer AP durations and a trend towards higher maximal upstroke velocities (Fig. 1e and Supplementary information, Fig. S10b). Monolayer cultures (500–600k) of CD36^hi^ CMs could also be electrically captured and stimulated to contract over a broader range of pacing cycle lengths (500–1500 ms) than CD36^mixed^ CMs (700–1500 ms). Consistent with this, we detected significantly increased mRNA levels of genes encoding subunits of the I_K1_ (KCNJ2: 2.69 ± 1.16 fold), I_Ca_ (CACNA1C: 3.30 ± 1.59 fold) and a trend towards increased I_Na_ (SCN5A: 3.27 ± 1.23 fold, *p* = 0.069). The gene encoding the I_f_ subunit (HCN1: 0.29 ± 0.12 fold), the current mainly responsible for automaticity, was significantly reduced (Supplementary information, Fig. S10c). Independent assessments of H7-derived CMs from two laboratories confirmed that the average frequency of cytosolic Ca^2+^ release was significantly decreased (Fig. 1f, Supplementary information, Fig. S11a–c). The amplitude of Ca^2+^ transients was also increased, but the time-to-peak and half decay time were either unchanged or modestly increased in CD36^hi^ *versus* CD36^lo^ CMs isolated from the same cultures (Fig. 1f, Supplementary information, Fig. S11c, d and movies S1 and 2). Genes important for Ca^2+^ handling, such as PLN and CASQ2, were significantly increased in CD36^hi^ CMs by 1.83 ± 0.38 and 2.64 ± 0.63 fold, respectively (Supplementary information, Fig. S11e).

The major differences between CD36^hi^ and CD36^lo^ CMs were predicted from RNAseq and gene ontology (GO) analyses to involve mitochondrial metabolism (Fig. 1g, Supplementary information, Tables S2 and 3). CD36^hi^ CMs were characterized by an increased prevalence of mitochondrial and oxidative phosphorylation (OXPHOS)-associated genes (Fig. 1g, Supplementary information, Fig. S12a). Of the 67 OXPHOS transcripts detected by RNA-seq, 33 (49%) were significantly and positively correlated with CD36 (Supplementary information, Fig. S12b, Table S4). Mitochondrial proteins from Complexes II (SDHB) and III (UQCRC2), and ACADVL were elevated in CD36^hi^ CMs (Supplementary information, Fig. S12c, d). Transcripts from non-mitochondrial glycolysis associated genes (e.g., HK2 and ENO2) were largely unchanged and non-correlative. In contrast, CD36^lo^ CMs showed significant increases in mRNAs encoding cell adhesion and ECM proteins (Supplementary information, Tables S2 and 3). Metabolically, CD36^hi^ CMs had increased mitochondrial function relative to CD36^lo^ CMs. Mitochondrial mass was increased, and these organelles adopted a more elongated and well-aligned staining pattern (Fig. 1h, i). CD36^hi^ CMs also had a >54% increase in mitochondrial membrane potential (Δψm) relative to CD36^lo^ CMs (Fig. 1i, j). ATP production was enhanced, and the cellular uptake of a fluorescently labeled C16-palmitate analog was higher in CD36^hi^ *versus* CD36^lo^ CMs (Fig. 1k, l). When cultured in nutrient-deficient media lacking fatty acids, the Δψm were similar, but when supplemented with oleic acid, the Δψm in CD36^hi^ CMs (43.0%) increased more significantly when compared with CD36^lo^ CMs (22.9%) (Fig. 1m, Supplementary information, S13) consistent with improved β-oxidation. These phenotypic and metabolic traits of CD36^hi^ CMs were maintained across diverse hPSC lines (Supplementary information, Fig. S14). Importantly, both hESC- and hiPSC-derived CD36^hi^ CMs displayed a more metabolically mature phenotype with decreased experimental variability relative to CD36^mixed^ cells in terms of Δψm, ATP production, fatty acid uptake and the %MLC2V^+^CMs (Supplementary information, Fig. S15).

We investigated whether electrical stimulation, which promotes electrophysiological and structural maturation, or activation of the PPARα signaling pathway, which promotes a more advanced mitochondrial phenotype,^8^ could up-regulate CD36. Electrical stimulation failed to up-regulate CD36 or to significantly affect the mitochondrial content and Δψm (Supplementary information, Fig. S16a, b). Electrically stimulated CMs gated for high CD36 expression, however, exhibit a greater mitochondrial content and Δψm compared to either CD36^mixed^ or CD36^lo^ CMs (Supplementary information, Fig. S16c). Application of the PPARα agonist, WY-14643, significantly increased the proportion of cells with CD36 on the cell surface (Supplementary information, Fig. S16d). These results are consistent with publicly available data, which show that electrical stimulation does not increase the mRNA level of CD36,^9^ whereas application of fatty acids and HIF-1α inhibition (together with PPARα activation and postnatal factors) promote mitochondrial maturation^10^ and upregulate CD36 mRNA levels (Supplementary information, Fig. S16e). Thus, CD36 is a marker of cardiac mitochondrial/metabolic maturation, and CD36 isolation can be combined with maturation protocols to immunodefine and isolate CMs with enhanced mitochondrial function.

Mitochondria-rich CD36^hi^ CMs show enhanced sensitivity to oxidative stimuli compared to CD36^mixed^ and CD36^lo^ CMs. The viability of unsorted CMs was largely unaffected by 10–250 µM hydrogen peroxide (H_2_O_2_) for treatment periods of 1 or 3 h. In contrast, treatment with 100 µM H_2_O_2_ for 30 min significantly reduced the viability of CD36^hi^, but not of CD36^mixed^ and CD36^lo^ CMs. CD36^hi^ CMs also exhibited more damage in terms of a decline in Δψm, enhanced production of reactive oxygen species (ROS), and an increase in the proportion of cells with nuclear condensation (Supplementary information, Figs. S17–S19). Using a more complex hypoxia (H) and re-oxygenation (R) oxidative stress injury model, all CD36 subpopulations experienced a decrease in viability and demonstrated hypoxic damage (i.e., elevated ROS production and nuclear condensation, reduced Δψm), which was exacerbated by reoxygenation; but these changes and the production of lactate dehydrogenase (LDH) were significantly more pronounced in CD36^hi^ CMs (Supplementary information, Fig. S20).

Doxorubicin (Dox), a commonly used anticancer drug, induces severe and irreversible cardiotoxicity through mitochondrial dysfunction and oxidative stress. Dox is toxic to both mixed and sorted CMs from hESCs and hiPSCs; however, CD36^hi^ CMs are more sensitive to Dox-induced damage than either CD36^lo^ or CD36^mixed^ CMs (Fig. 1n, Supplementary information, Fig. S21). Furthermore, dexrazoxane, the only FDA-approved drug that protects against doxorubicin-induced cardiotoxicity, is reported to be detrimental to conventionally-grown hPSC-CMs.^11^ Unexpectedly, CD36^hi^, but not CD36^mixed^ or CD36^lo^ CMs, responded positively to the protective effects of dexrazoxane (200 µM) (Fig. 1o). Indeed, pre-treatment with dexrazoxane significantly improved the viability and the Δψm of Dox-treated CD36^hi^ CMs. Conversely, N-acetylcysteine, which is effective in mouse models of Dox-induced cardiotoxicity but *unprotective* in human clinical trials, did not have any significant impact on CD36^hi^ CM viability except at non-physiological doses (10 mM). However, it did protect Dox-treated CD36^lo^ CMs at a dose of 0.1 mM, consistent with results from in vitro animal experiments and in non-sorted hPSC-CMs^11^ (Fig. 1o).

In conclusion, we report the presence of a cell surface marker that can immuno-define more mature hPSC-CMs and reduce experimental variability. These properties make CD36^hi^ CMs well suited for disease modelling and drug screening in general, and for studies of Dox-induced cardiotoxicity in particular. The CD36^hi^ CM model is an improvement over existing animal models, which fail to eliminate false positives (e.g., N-acetylcysteine), and to existing hPSC-CM models, which fail to respond to authenticated cardioprotective agents (dexrazoxane). Since mitochondrial dysfunction contributes to a broad range of adult diseases (e.g., arrhythmogenic right ventricular dysplasia/cardiomyopathy), injuries (e.g., ischemia/reperfusion) and cardiotoxicities (e.g., Dox), the study of hPSC-CD36 CM subpopulations will advance efforts to understand and promote cardiac mitochondrial maturation processes in vitro and provide the impetus for informed investigations of adult cardiac syndromes that involve mitochondrial dysfunction.

## Supplementary information

Supplementary Information

Supplementary Movie S1 Ca^2+^ transient signals in CD36^hi^ CMs

Supplementary Movie S2 Ca^2+^ transient signals in CD36^lo^ CM
