# Supplementary material for: The cell surface marker CD36 selectively identifies matured, mitochondria-rich hPSC-cardiomyocytes
Source: Cell Res. 2020 Mar 10;30(7):626–9. doi: 10.1038/s41422-020-0292-y (PMC7343859; doi:10.1038/s41422-020-0292-y)
Supplement: Supplementary file 1 — Supplementary Information [file 41422_2020_292_MOESM1_ESM.pdf]

Supplemental Methods

The Cell Surface Marker CD36 Selectively identifies Matured, Mitochondria-rich hPSC-Cardiomyocytes

Ellen Ngar-Yun Poon, Xiao-ling Luo, Sarah E. Webb, Bin Yan, Rui Zhao, Stanley Chun Ming Wu, Yong Yang, Peng Zhang, Huajun Bai, Jiaofang Shao, Ching Man Chan, Godfrey Chi-Fong Chan, Suk Ying Tsang, Rebekah L. Gundry, Huang-Tian Yang, Kenneth R. Boheler

hPSC culture and cardiomyocyte differentiation

Undifferentiated hESCs (H7, H9) and hiPSCs (MDI-C16,[1] JHU001[2]) were maintained on Matrigel (BD Biosciences, Sparks, MD)-coated surfaces at 37°C and 5% CO_2 in E8 medium plus supplements (Thermo Fisher Scientific, Waltham, MA), and differentiated to CMs as described previously, but with modifications.[3-5] Initially, hPSCs were plated at a density of 70-150k hPSCs (depending on the line) in E8 medium into individual wells of a 6-well plate for 4 days as previously described. To initiate differentiation, the E8 medium was changed to RPMI/B27 medium lacking insulin. CHIR99021 (6 μM; Selleckchem, Houston, TX) was added to the cells from days 0 to 2, followed by the addition of IWR-1 (10 μM; Selleckchem) from days 3 to 5 for H7 and JHU001 cells, and from days 4 to 6 for H9 and MDI-C16 cells. After day 7, cultures were maintained in RPMI/B27 medium containing insulin (Thermo Fisher Scientific), which was changed twice a week. For the induction of atrial-like cells, hPSC cultures were supplemented with 2 or 5 μM all-*trans* retinoic acid (RA), depending on the line, for 48

to 72 hours starting at day 3 of differentiation.[6] Cultures were passaged using 0.05% Trypsin/EDTA (Thermo Fisher Scientific) every 1-2 weeks. In some experiments, hPSC-derived CM cultures were maintained in RPMI/B27 medium without glucose but supplemented with 4 mM lactose (Sigma Aldrich Corp., St Louis, MO) for 4-7 days to remove any potentially contaminating non-CMs.[7] No effects were observed with respect to either CD36 expression or cellular behaviour when the cells were treated with or without lactose. Thus, results from cells with and without lactose treatment were combined for analysis.

Identification of cell surface proteins using the Cell Surface Capture (CSC) Technology

CMs (~50 million / experiment) that were >95% TNNT2 positive were taken through the CSC Technology workflow, as reported previously[4,8-10] with modifications. In brief, CMs were washed with ice cold labelling buffer (phosphate-buffered saline (PBS) pH 6.5, 0.1% fetal bovine serum (FBS)), followed by treatment with labelling buffer containing 1 mM sodium metaperiodate (Pierce, Rockford, IL) for 15 min at 4°C. Cells were washed 2x with cold labelling buffer, and then incubated with labelling buffer containing 2.5 mg/mL biocytin hydrazide (Biotium, Hayward, CA) for 1 h at 4°C on a rocker. The biocytin solution was removed, and the cells were washed 2x with cell labelling buffer. Hypotonic lysis buffer (10 mM Tris pH 7.5 containing 0.5 mM $MgCl_2$) was added to the plates for 10 min, followed by cell scraping. Using a serological pipette, the cells were transferred to an *M tube* and lysed 2x using a GentleMACS dissociator, as described.[7] Combined cell lysates were centrifuged at 1000 x *g* for 10 min at 4°C to remove debris. An equal volume of membrane preparation buffer (280 mM sucrose, 50 mM MES pH 6.5, 450 mM NaCl, 10 mM $MgCl_2$)

was added to the supernatant and the mixture was transferred to ultracentifuge tubes prior to centrifugation at 210,000 x *g* for 4 h at 4°C to collect the membrane proteins. The supernatant was discarded, and 25 mM Na_2CO_3 (200 µL) was added to the pellet. This was vortexed in a thermomixer at 750 rpm for 30 min at 4°C. Hypotonic lysis buffer was added, and the entire contents were centrifuged as described above for 1 h at 4°C. After discarding the supernatant, the membrane protein pellet was resuspended in 100 mM NH_4HCO_3 (300 µL), followed by the addition of 1% (v/v) Rapigest (40 µL, Waters, Milford, MA) and then 100 mM Tris(2-carboxyethyl) phosphine (25 µL, Sigma Aldrich Corp.). This was vortexed for 10 min at 25°C to allow sufficient time for protein reduction. Protein alklylation was performed by the addition of iodoacetamide (10 mM, final) for 30 min, after which the sample was incubated with 1 µg glycerol-free endoproteinase Lys-C (Calbiochem, San Diego, CA) at 37°C for 4 h, followed by 2 overnight applications of 20 µg proteomics grade trypsin (Promega Corp., Madison, WI) at 37°C. Enzymes were then inactivated by the addition of one drop of phosphoric acid (Sigma Aldrich Corp.). The resulting peptide mixture was incubated with end-over-end rotation after applying to 450 µL pre-washed UltraLink Immobilized Streptavidin PLUS (Pierce) loaded onto a MoBiCol column for 1 h at 25°C. Streptavidin beads on the column were sequentially washed with 0.5% Triton X-100 in 100 mM NH_4HCO_3, 5 M NaCl, 100 mM Na_2CO_3, 80% isopropanol and then 100 mM NH_4HCO_3, to remove nonspecific peptides and lipids. The beads were resuspended in 100 mM NH_4HCO_3 and 500 units glycerol-free endoproteinase PNGaseF (New England Biolabs, Ipswich, MA), and incubated at 37°C for 16 h with end-over-end rotation to release the peptides from the beads. Collected peptides were desalted and concentrated using a C18 UltraMicroSpin™ column (Nest Group, Southborough, MA) according to the manufacturer's instructions. Mass spectrometry data were acquired

using an LTQ Orbitrap Velos (Thermo Fisher Scientific) and data analyses were completed as previously described.[4] Cell surface proteins were defined as those in the dataset identified by at least one peptide containing a deamidation at the asparagine residue within the conserved sequence motif for N-glycosylation (NxS/T/C, where X≠P).

Cell surface proteins were then analysed using the DAVID bioinformatics database[11] to reveal those that were present on the membrane (UP_KEYWORDS_Membrane), on the extracellular matrix (GO: GO:0005886), or that were GPI-anchored (UP_KEYWORDS_GPI-anchor). CD molecules were identified by comparison with http://www.biolegend.com/media_assets/support_resource/Human_CD_Molecules.pdf. Cell type/line-specific expression was established by comparison with the Cell Surface Protein Atlas.[12] Proteins that were present in <20% (10/47) of the cell types/lines examined were considered to show cardiac-restricted expression.

Flow cytometry and fluorescence activated cell sorting

Differentiated CM cultures were analysed by flow cytometry essentially as described using antibodies to TNNT2 (see Table S5).[13] Cells were rinsed with DMEM/F12 and dissociated with accutase (3 mL/10 cm dish) for 20-30 min (Thermo Fisher Scientific) at 37°C. CMs were transferred into a conical tube containing DMEM/F12, and the pellet was collected by centrifugation at 300 x *g* for 4 min at 4°C. Cells were washed 2x with wash buffer (1% FBS in Dulbecco's PBS (DPBS) (no Mg^{2+}, no Ca^{2+}) pH 7.4) and then placed in blocking solution (DPBS + FBS supplemented with 10% goat serum (Thermo Fisher Scientific) and 10 µM Y27632) for 15 min at 4°C. The cells were then incubated with the primary antibody (CD36-APC, SIRPA-PE,[14]

TNNT2 or isotype controls, see Table S6) for 1 h at 4°C, after which they were washed twice with wash buffer. Isotype controls were used at the same concentration as the primary antibody and undifferentiated hPSCs served as negative controls. Where necessary, cells were washed twice with wash buffer and then they were incubated with the appropriate secondary antibody for 1 h at 4°C, followed by two more washes.

For analysis by flow cytometry, immunostained cells were resuspended in wash buffer and passed through a cell strainer prior to analysis on a BD FACSCanto II or BD Fortessa (BD Biosciences, San Jose, CA) flow cytometer. A minimum of 10,000 events were acquired and data were analysed with the FlowJo software. The percent positive cells was based on gated cells with a background contribution of <2%.

Sorting of CMs was performed on differentiation day 45 ± 5 days unless otherwise noted, using cells suspended in cell resuspension buffer [Hank's buffered saline solution (HBSS) supplemented with 5% FBS, Y27632 (10 µM; Selleckchem), HEPES (25 mM; Thermo Fisher Scientific)]. Sorting was accomplished using a BD FACS ARIA SORP, Influx sorter (BD Biosciences) or a SH800 FACS sorter (Sony Biotechnology Inc., San Jose, CA). Sorting was based on CD36 detection, and CD36^{hi} and CD36^{lo} CMs were defined as cells with the top and bottom 25-30 percentile of CD36-APC staining, respectively. Cells were simultaneously gated for the presence of CD172a (SIRPA) to ensure that the cell sorts consisted mainly of CMs.[14] For CD36^{mixed} cells, CD172a^{+} CMs were sorted without CD36 gating. Three subpopulations (i.e., CD172a^{+}/CD36^{mixed}, CD172a^{+}/CD36^{hi}; and CD172a^{+}/CD36^{lo}) were used for all RNA-seq and subsequent experiments, unless otherwise indicated. Cells were collected in RPMI medium supplemented with B27+insulin, 10 µM Y27632, 2x penicillin/streptomycin, and 2% FBS. Sorted cells were plated onto geltrex-coated surfaces and were analysed 1-3 weeks after seeding.

RNA sequencing and analysis

Total RNA was extracted using an RNeasy micro kit (Qiagen, Hilden, Germany), according to the manufacturer's protocol. cDNA libraries were prepared using the KAPA Stranded mRNA-Seq Kit. One hundred nanograms of total RNA were used as the starting material. The manufacturer's protocol was followed throughout library preparation. In brief, Poly-A containing mRNA was collected by using poly-T oligo-attached magnetic beads. The purified mRNA was broken down into short fragments and was applied as template to synthesize the first-strand cDNA by using random hexamer-primers and reverse transcriptase. In the second strand cDNA synthesis, the mRNA template was removed and a replacement strand was generated to form blunt-end double-stranded (ds) cDNA. The ds cDNA underwent 3' adenylation and indexed adaptor ligation, after which the adaptor-ligated libraries were enriched by 15 cycles of polymerase chain reaction (PCR). The libraries were denatured and diluted to an optimal concentration, after which they were applied to the HiSeq PE Cluster Kit v4 with cbot for cluster generation on the flow cell. Illumina HiSeq SBS Kit v4 was used for Pair-End 101 bp sequencing. Sequencing was performed in the HKU Genome Centre.

The raw data produced by RNA-seq experiments were aligned to the human genome using TopHat.[15] The read counts at the gene level defined by GENCODE annotation were extracted using the HTSeq tool. The counting table was then used as input to identify differentially expressed genes using DESeq2.[16] A 1.4-fold difference in transcript abundance among CD36^{hi}, CD36^{lo} and CD36^{mixed} CMs were considered as differentially expressed ($p<0.05$). Significantly enriched gene sets among the various categories of biological functions, were identified from Gene Ontology cellular

components, biological processes and KEGG Pathways. The association between CD36 and genes involved in oxidative phosphorylation (GO:0006119) was examined by Pearson correlation analysis. A Pearson correlation coefficient of gene expression among $CD36^{hi}$, $CD36^{lo}$ and $CD36^{mixed}$ CMs was also calculated.

Quantitative real-time PCR (qRT-PCR)

Total RNA was extracted using Trizol (Thermo Fisher Scientific), according to the manufacturer's protocol. cDNA was synthesized with the QuantiTect Reverse Transcription Kit (Qiagen). qRT-PCR was performed as previously described.[17] RNA levels were normalized to GAPDH and then further normalized to the control sample, in order to control for biological variations among different sets of samples. Three technical replicates were run per sample. A Student's t-test was used to determine statistical significance, and $p<0.05$ was considered to be statistically significant.

Immunofluorescence staining

Cultures of *in vitro* differentiated CMs were dissociated with accutase and plated onto geltrex-coated glass coverslips in RPMI/B27 medium containing insulin (Thermo Fisher Scientific). Samples were fixed with PBS containing 4% paraformaldehyde for 15 min at room temperature, and then permeabilized in PBS containing 1% Triton X-100 at room temperature for 10 min. After blocking with 10% normal donkey serum in PBS at room temperature for 1 h, samples were incubated with primary antibodies (Table S5) in PBS containing 1% donkey serum at 4°C overnight. After washing, secondary antibodies (Thermo Fisher Scientific) were applied for 1 h at room temperature in the dark. The coverslips containing the cells were then mounted onto glass slides using Prolong diamond mounting medium

(Thermo Fisher Scientific). The samples were imaged with a Nikon Eclipse TiS, Leica DMi8 microscopes, or with a Carl Zeiss LSM 700 confocal microscope.

Mitochondrial copy number quantification

Genomic DNA (gDNA) extraction was performed using the MasterPure-Complete DNA and RNA Purification Kit (Epicentre, Madison, WI), according to the manufacturer's instructions, and with RNase treatment. The mitochondrial content is expressed as the mitochondrial DNA (mtDNA) to nuclear DNA (nDNA) ratio, where the nDNA copy number per cell is considered to be constant. Changes in the ratio were attributed to be due to changes in the content of mtDNA. Real-time amplifications of the mitochondrial genes, NADH dehydrogenase subunit I (ND1) and NADH dehydrogenase subunit V (ND5), and the nuclear gene, GAPDH, were performed as described above, in triplicate. Gene expression was normalized to GAPDH and this was further normalized to that of the control sample to control for biological variations among the different sets of samples. A Student's t-test was used to determine statistical significance, and $p<0.05$ was considered to be statistically significant.

Mitochondrial membrane potential (Δψm) measurements

Mitochondrial membrane potential (Δψm) was measured using the following mitochondrial dyes: tetramethylrhodamine, ethyl ester (TMRE), tetramethylrhodamine, methyl ester (TMRM) and MitoTracker Green FM (MTG). TMRE and TMRM are potentiometric dyes whose fluorescent intensity is proportional to the mitochondrial potential,[18] whereas MTG stains the mitochondria in a Δψm-independent manner.[19] The TMRE:MTG and TMRM:MTG ratios are indicators of Δψm. For TMRE/MTG staining, CMs were plated into geltrex-coated surfaces in RPMI/B27 medium. Cells

were incubated with TMRE (25 nM; Thermo Fisher Scientific) and MTG (200 nM; Thermo Fisher Scientific) for 30 min at 37ºC, after which they were washed with DPBS and imaged with a Synergy H1 plate-reader (Biotek, Winooski, VT) using absorption/emission wavelengths of 530 nm/590 nm for TMRE and 485 nm/530 nm for MTG. To assess substrate utilisation, CMs were switched one day prior to the assay, to nutrient-deficient XF media lacking glucose and fatty acids (Agilent Technologies, Santa Clara, CA). On the day of the assay, cells were incubated with TMRE and MTG as described above. Oleic acid conjugated to bovine serum albumin (Oleic-acid-BSA; 0.1 mM; Sigma Aldrich Corp.) was applied. For confirmation, $\Delta\psi m$ was also measured using TMRM/MTG staining. For these assays, CMs were plated into glass chambers (Ibidi) in RPMI/B27 medium and stained with TMRM (25 nM; Thermo Fisher Scientific) and MTG (200 nM; Thermo Fisher Scientific) for 30 min at 37ºC. Samples were imaged with a Zeiss Axio Observer microscope, using the red and green channels. Fluorescence intensities were quantified using ImageJ, and $\Delta\psi m$ was calculated as the ratio of red/green fluorescence.

ATP measurements and fatty acid uptake assay

ATP levels were measured using the CellTiter-Glo luminescent cell viability assay (Promega Corp.), according to the manufacturer’s instructions.

Fatty acid uptake was monitored using BODIPY FL C16 (Thermo Fisher Scientific), which is a fluorescently labeled analog of palmitic acid. Human PSC-CMs were incubated with 10 µM BODIPY FL C16, 3% BSA in RPMI media for 1 h at 37ºC. Cells were then washed with DPBS and the fluorescence intensity was measured using the Synergy H1 plate-reader with excitation and emission wavelengths of 485 nm and 530 nm, respectively.

Assessment of cellular parameters

Morphology:

To determine the proportion of binucleated cells, samples were stained with anti-α-actinin antibodies to identify and define individual CMs. Nuclei were revealed by DAPI staining and the number of nuclei per cell was counted under a fluorescence microscope. An average of >100 cells were counted per batch and five batches of CMs were examined. To confirm that cells with two nuclei represent binucleated cells and not cells undergoing cell division, samples were stained with Ki67, which specifically labels proliferating cells, and day 25 and/or undifferentiated hPSCs were used as positive controls.

Sarcomeric score:

To determine the degree of sarcomeric organization, samples were stained with anti-α-actinin antibodies and individual cells were evaluated based on a semi-quantitative system. A numerical score was assigned to each cell in a blinded study based on its sarcomeric arrangement as indicated below:

4 Cells contained α-actinin + Z-bands parallel to each other.

3 Cells contained a mixture of α-actinin staining patterns, with both regular/parallel and irregular arrangements.

2 α-actinin staining was disorganised, with little parallel arrangement detected.

1 Punctate, non-linear α-actinin staining corresponding to z-bodies.

Numerical scores were tallied on >100 cells from 7 independent batches of CMs. Once scores were calculated, a final sarcomeric score was given to each group.

***Electrophysiology*:**

Cells on glass coverslips were placed in a chamber filled with Tyrode's solution [140 mM NaCl, 5.4 mM KCl, 1.8 mM CaCl2, 1 mM MgCl2, 10 mM D-glucose, 10 mM HEPES, pH 7.4 (adjusted by NaOH)] at 34°C. Recordings of spontaneous action potentials (AP) were acquired in current-clamp mode of whole-cell patch clamp technique using an Axopatch 200B amplifier (Molecular Devices, Sunnyvale, CA, USA) at 2 kHz. Microelectrodes (4–7 MΩ) pulled from P-97 puller (Sutter Instrument, Novato, CA, USA), and were filled with 110 mM K+ aspartate, 20 mM KCl, 1 mM MgCl2, 1 mM EGTA, 10 mM HEPES, 5 mM Mg-ATP, 0.1 mM Na-GTP, 5 mM Na2-phospocreatine, pH 7.3 (adjusted by KOH). Data were analyzed using CAPA software (SCCE UG, Essen, Germany). Default setting of the software was used. Optical mapping, graciously performed by Dr. Blazeski, were as previously described.[20]

Action Potentials (AP) from control and retinoic acid-treated hPSC-CMs were recorded using the EPC-10 amplifier (Heka Electronics, Bellmore, NY) in current-clamp mode. Cells attached to coverslips were put in a temperature-controlled (35°C) recording chamber which was filled with the bath solution (140 mM NaCl, 5 mM KCl, 1.8 mM $CaCl_2$, 2 mM $MgCl_2$, 10 mM D-glucose, and 10 mM HEPES, pH 7.4). The pipette electrode (3~6 MΩ) was filled with a solution containing 20 mM KCl, 110 mM K-asparate, 1 mM $MgCl_2$, 1 mM EGTA, 10 mM HEPES, 0.1 mM NaGTP, 5 mM MgATP, and 5 mM Na2-phospocreatine (pH 7.3, adjusted with KOH).

To characterize the cardiomyocyte subtype, the beating rate (frequency), maximum diastolic potential (MDP), peak voltage (peak), amplitude (AMP), maximal rate of depolarization (dV/dtmax), and AP duration (APD) at different levels of repolarization (i.e., 90%, 80%, 70%, 50%, 40%, and 30%) were analyzed. Multiple

criteria were used to distinguish the AP phenotypes as described.[21,22] The cells were classified into ventricular- or atrial- like based on the more negative MDPs, higher dV/dtmax values and larger amplitudes when compared with nodal-like cells. Ventricular-like cardiomyocytes were characterized by a rapid AP upstroke, a long plateau phase and accelerated repolarization. Thus, cells with an APD90/APD50 ratio < 1.4 and and APD30-40/APD70-80 ratio >1.5 were considered to be ventricular-like, whereas atrial-like cardiomyocytes were characterized by the absence of a prominent plateau phase, as well as an APD90/APD50 ratio > 1.7 and an APD30-40/APD70-80 ratio <1.5. And cells with a slower AP upstroke, a prominent phase 4 depolarization, and an APD90/APD50 ratio between 1.4-1.7 were categorized as nodal-like.

Measurement of calcium transients:

Cells were loaded with 2 μM Fluo-4 AM fluorescent Ca^{2+} indicator (Thermo Fisher Scientific) dissolved in the extracellular bath solution (140 mM NaCl, 5 mM KCl, 1.8 mM $CaCl_2$, 2 mM $MgCl_2$, 10 mM D-glucose, 10 mM HEPES, pH 7.4) for 15 min at 37°C, washed three times with the same bath solution and then incubated in this solution at 37°C for 15 min before use. Global Ca^{2+} transients (CaTs) were monitored using a confocal laser scanning microscope (LSM 710, Carl Zeiss) with a ×40 oil objective in line scan mode. The fluorescence intensity was excited with wavelength 488 nm and the emission was collected with wavelength > 493 nm. Each line was collected with an interval of 10 ms. Images were analyzed using IDL software.

Cells attached to glass coverslips were washed with Tyrode's solution which contained 140 mM NaCl, 5.4 mM KCl, 1.8 mM $CaCl_2$, 1 mM $MgCl_2$, 10 mM D-glucose, 10 mM HEPES, pH 7.4 (adjusted with NaOH) and then incubated with 5 μM Fluo-4 AM and 0.02% (w:v) pluronic acid F-127 (Thermo Fisher Scientific) in Tyrode's solution for 20 min in the dark at 37°C. Global CaTs were monitored with an Olympus Fluoview

FV1000 confocal microscope at 60 Hz using the X-Y-T scan mode. Parameters including amplitude, frequency, time-to-peak, and 50% decay time were manually analyzed using the Origin 6.1 software (OriginLab, Northampton, MA,) from the average of 8 CaTs/cell.[23]

Electrical Stimulation

The electrical stimulation platform contained 6-well-plates for cell culture and a multi-mode stimulator (IonOptix C-PACE EP, USA). Briefly, hPSC-CMs on day 14 were plated and assigned to control or experimental group with electrical stimulation (ES). An intensity training regime was adopted in the ES group, where the stimulation started at 6 mV intensity, 1 ms in duration, 2 Hz and the frequency was increased by 0.33 Hz per day throughout the week.

After one-week stimulation, CMs in each group were incubated with the 100 nM TMRE and 100 nM MitoTracker Green (MTG) probes on plates for 30 minutes at 37°C. The cells were then dissociated and stained with CD36 antibody. The mitochondrial content and membrane potential were measured using MTG and TMRE staining, and the expression level of CD36 were analyzed by flow cytometry.

Induction of oxidative stress in hESC-CMs

CMs were sorted on differentiation day 45 ± 5 days and plated into geltrex-coated 96-well plates at a density of ~20,000 cells/well. Plated cells were allowed to recover for 1-3 weeks. Two experimental conditions were tested: 1) cells were treated with 100 µM H_2O_2 in DMEM medium containing 10% FBS for 30 min, followed by analysis; or 2) cells were subjected to hypoxia and reoxygenation[24]. For the latter, the

culture medium was replaced with glucose-free DMEM basal media followed by induction of hypoxia (i.e., < 0.1% O_2) at 37°C and 5% CO_2 for 4 h without nutrient supplementation, in an oxygen-controlled incubator (Ruskinn Technology Ltd., Bridgend, Wales). The cells were then reoxygenated with RPMI 1640 supplemented with B27/RPMI and cultured under normoxic conditions for 1 h. Control cells were kept for equivalent time periods under normoxic standard conditions in RPMI/B27+insulin medium.

Induction of cardiotoxicity in hESC-CMs

CMs were treated with 1 µM doxorubicin for 24 h in DMEM low glucose media (Thermo Fisher Scientific). In the cardioprotection assays, CMs were pre-treated with various concentrations of dexrazoxane or N-acetylcysteine for 1 h, followed by co-treatment with 1 µM doxorubicin for 24 h.

Mitochondrial superoxide measurements

Following the induction of oxidative stress (or controls), mitochondrial superoxide (O_2^-) levels were assayed using the MitoSOX™ Red reagent (Thermo Fisher Scientific), according to the manufacturer's instructions. CMs were treated with 2.5 µM MitoSOX™ Red reagent for 10 min at 37ºC. After washing, samples were imaged with a Nikon Eclipse TiS microscope, using the red channel. Fluorescence intensities were quantified using ImageJ.

Viability and Lactate dehydrogenase (LDH) measurements

Viability was determined using two independent methods. The Presto blue assay (Thermo Fisher Scientific) was performed according to the manufacturer's

instructions by the addition of Presto blue reagent to CMs for 1 h at 37ºC. The intensity of fluorescence was measured using the Synergy H1 plate-reader with excitation and emission wavelengths of 560 nm and 590 nm, respectively. The MTT assay (Sangon Biotech, Shanghai, China) was performed on CMs incubated with 0.5 mg/mL MTT at 37°C for 4 h. The formazan product was dissolved with DMSO (Sigma Aldrich Corp.) and the absorbance at 570 nm was measured with the Synergy H1 plate-reader.

LDH release (C0016, Beyotime Biotechnology, Shanghai, China) was measured using 80 μL of supernatant collected from both the hypoxia and the reoxygenation stages of oxidative stress. Using a 96-well plate format, 40 μL of substrate was added to the samples. Incubations were performed at room temperature without light exposure for 30 min, after which time the absorbance was recorded at 490 nm using the Synergy H1 plate-reader.

Determination of intra- and inter-line variabilities by flow cytometry

To examine intra- and inter-line variabilities, hESC- and hiPSC-CMs were stained with TMRE and MTG, or with BODIPY FL C16, as described above. The CMs were then dissociated with accutase and immunolabeled with the anti-CD36 antibody as described above (Flow Cytometry section). A uniform gating threshold for CD36 positivity was applied to ensure similar CD36 levels in gated populations. DAPI (2 μM) was applied to aid in the exclusion of any dead cells from the analysis. The Δψm and fatty acid uptake were determined by flow cytometry and was calculated as the ratio between TMRE and MTG staining (PE-A/FITC-A) or as BODIPY FL C16 staining (FITC-A).

Statistical Analysis

Statistical comparisons were performed using paired/unpaired Student's t-test for comparisons between two groups and ANOVA among three groups. $p<0.05$ was considered to be statistically significant. Data are presented as mean ± S.E.M.

References

1 He, J. *et al.* Generation of Induced Pluripotent Stem Cells from Patients with COL3A1 Mutations and Differentiation to Smooth Muscle Cells for ECM-Surfaceome Analyses. *Methods Mol Biol* **1722**, 261-302 (2018).

2 Ong, C. S. *et al.* Creation of Cardiac Tissue Exhibiting Mechanical Integration of Spheroids Using 3D Bioprinting. *Journal of visualized experiments : JoVE* (2017).

3 Bhattacharya, S. *et al.* High efficiency differentiation of human pluripotent stem cells to cardiomyocytes and characterization by flow cytometry. *Journal of visualized experiments : JoVE*, 52010 (2014).

4 Boheler, K. R. *et al.* A human pluripotent stem cell surface N-glycoproteome resource reveals markers, extracellular epitopes, and drug targets. *Stem cell reports* **3**, 185-203 (2014).

5 Wang, Y. *et al.* Nitric Oxide-cGMP-PKG Pathway Acts on Orai1 to Inhibit the Hypertrophy of Human Embryonic Stem Cell-Derived Cardiomyocytes. *Stem Cells* **33**, 2973-2984 (2015).

6 Lee, J. H. *et al.* Human Pluripotent Stem Cell-Derived Atrial and Ventricular Cardiomyocytes Develop from Distinct Mesoderm Populations. *Cell Stem Cell* **21**, 179-194 e174 (2017).

7 Tohyama, S. *et al.* Distinct metabolic flow enables large-scale purification of mouse and human pluripotent stem cell-derived cardiomyocytes. *Cell Stem Cell* **12**, 127-137 (2013).

8 Gundry, R. L. *et al.* The mouse C2C12 myoblast cell surface N-linked glycoproteome: identification, glycosite occupancy, and membrane orientation. *Molecular & cellular proteomics : MCP* **8**, 2555-2569 (2009).

9 Wollscheid, B. *et al.* Mass-spectrometric identification and relative quantification of N-linked cell surface glycoproteins. *Nat Biotechnol* **27**, 378-386 (2009).

10 Gundry, R. L. *et al.* A cell surfaceome map for immunophenotyping and sorting pluripotent stem cells. *Molecular & cellular proteomics : MCP* **11**, 303-316 (2012).

11 Huang da, W. *et al.* Systematic and integrative analysis of large gene lists using DAVID bioinformatics resources. *Nature protocols* **4**, 44-57 (2009).

12 Bausch-Fluck, D. *et al.* A mass spectrometric-derived cell surface protein atlas. *PLoS One* **10**, e0121314 (2015).

13 Waas, M. *et al.* Are These Cardiomyocytes? Protocol Development Reveals Impact of Sample Preparation on the Accuracy of Identifying Cardiomyocytes by Flow Cytometry. *Stem cell reports* **12**, 395-410 (2019).

14 Dubois, N. C. *et al.* SIRPA is a specific cell-surface marker for isolating cardiomyocytes derived from human pluripotent stem cells. *Nat Biotechnol* **29**, 1011-1018 (2011).

15 Trapnell, C. *et al.* TopHat: discovering splice junctions with RNA-Seq. *Bioinformatics* **25**, 1105-1111 (2009).

16 Love, M. I. *et al.* Moderated estimation of fold change and dispersion for RNA-seq data with DESeq2. *Genome Biol* **15**, 550 (2014).

17 Poon, E. N. *et al.* Integrated transcriptomic and regulatory network analyses identify microRNA-200c as a novel repressor of human pluripotent stem cell-derived cardiomyocyte differentiation and maturation. *Cardiovasc Res* **114**, 894-906 (2018).

18 Scaduto, R. C., Jr. *et al.* Measurement of mitochondrial membrane potential

using fluorescent rhodamine derivatives. *Biophys J* **76**, 469-477 (1999).

19 Pendergrass, W. *et al.* Efficacy of MitoTracker Green and CMXrosamine to measure changes in mitochondrial membrane potentials in living cells and tissues. *Cytometry. Part A : the journal of the International Society for Analytical Cytology* **61**, 162-169 (2004).

20 Blazeski, A. *et al.* Functional Properties of Engineered Heart Slices Incorporating Human Induced Pluripotent Stem Cell-Derived Cardiomyocytes. *Stem cell reports* **12**, 982-995 (2019).

21 Ma, J. *et al.* High purity human-induced pluripotent stem cell-derived cardiomyocytes: electrophysiological properties of action potentials and ionic currents. *Am J Physiol Heart Circ Physiol* **301**, H2006-2017 (2011).

22 Burridge, P. W. *et al.* Chemically defined generation of human cardiomyocytes. *Nature methods* **11**, 855-860 (2014).

23 Yang, H. T. *et al.* The ryanodine receptor modulates the spontaneous beating rate of cardiomyocytes during development. *Proc Natl Acad Sci U S A* **99**, 9225-9230 (2002).

24 Ng, K. M. *et al.* Cobalt chloride pretreatment promotes cardiac differentiation of human embryonic stem cells under atmospheric oxygen level. *Cell Reprogram* **13**, 527-537 (2011).

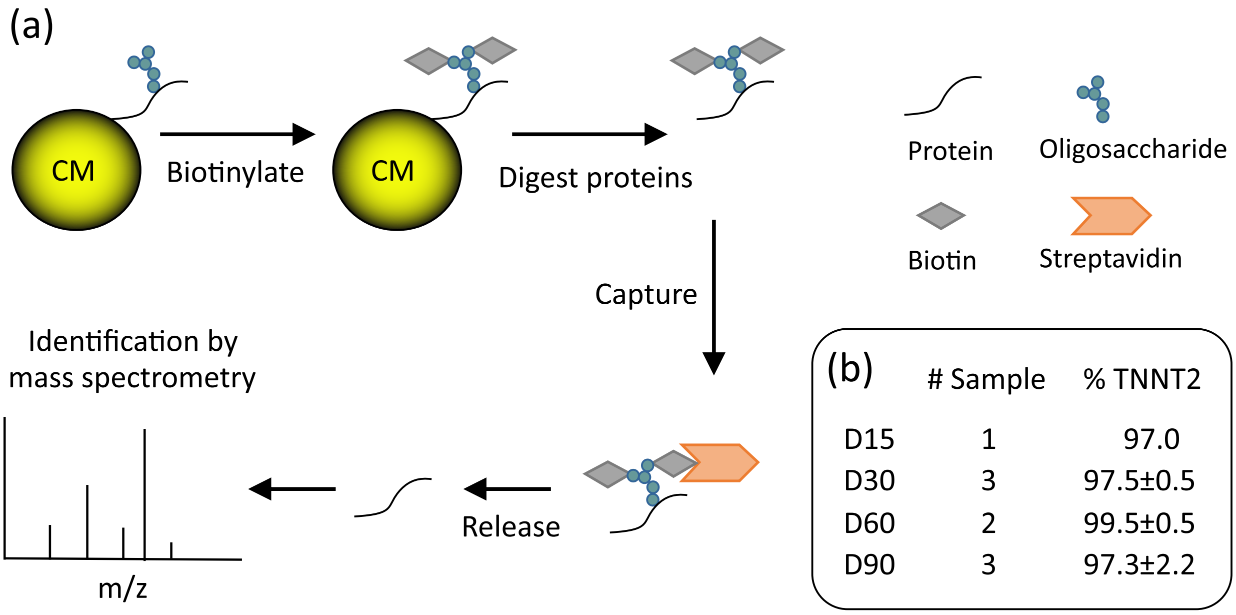

(b)

	# Sample	% TNNT2
D15	1	97.0
D30	3	97.5±0.5
D60	2	99.5±0.5
D90	3	97.3±2.2

Figure S1. (a) Schematic of cell surface capture (CSC) work flow. (b) Samples used for CSC experiments. D=Day of differentiation, #= number of samples used for experiment. The proportion of cardiac troponin T (TNNT2) positive cells (%TNNT2) was determined by flow cytometry using an anti-TNNT2 antibody, and data are expressed as mean±SEM.

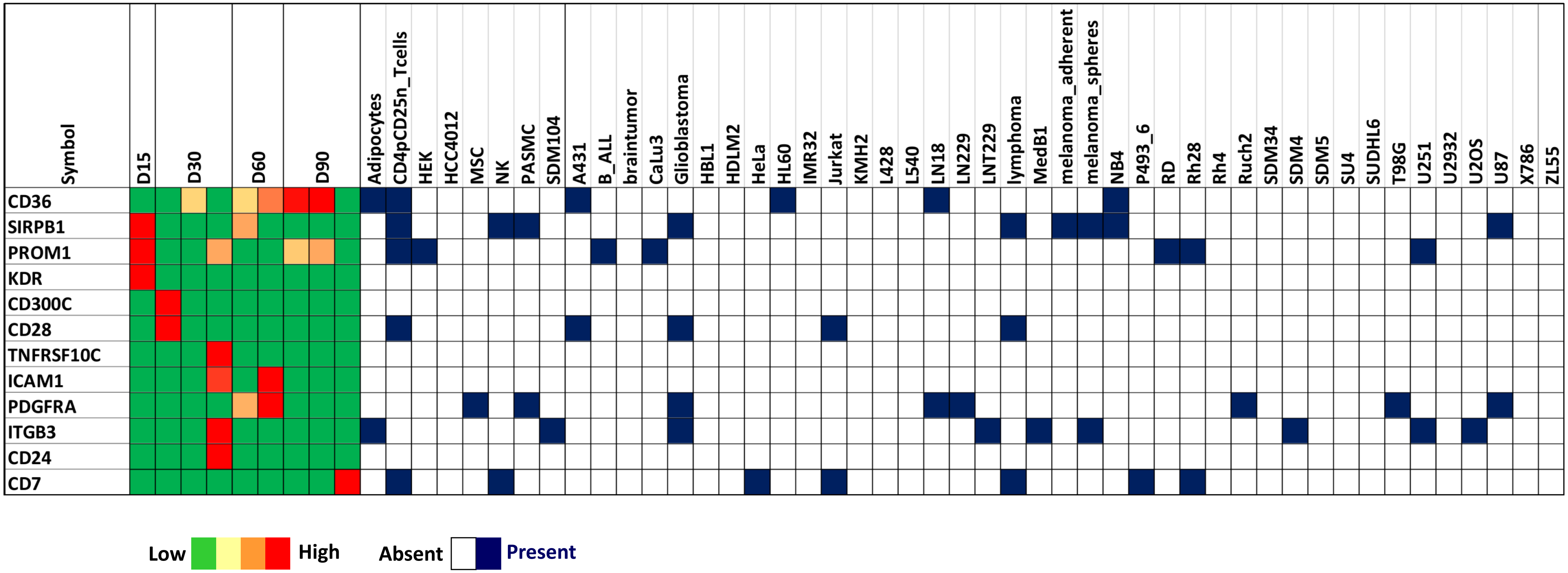

Figure S2. CD molecules identified by CSC technologies. In the colored graphic, the heat map shows normalized peptide counts identified by mass spectrometry of CD molecules in hESC-derived CMs at days (D)15, 30, 60 and 90. On the right is a blue and white heat-map showing the presence (blue) or absence (white) of these CD molecules in a panel of cell lines based on CSPA CSC protein database.

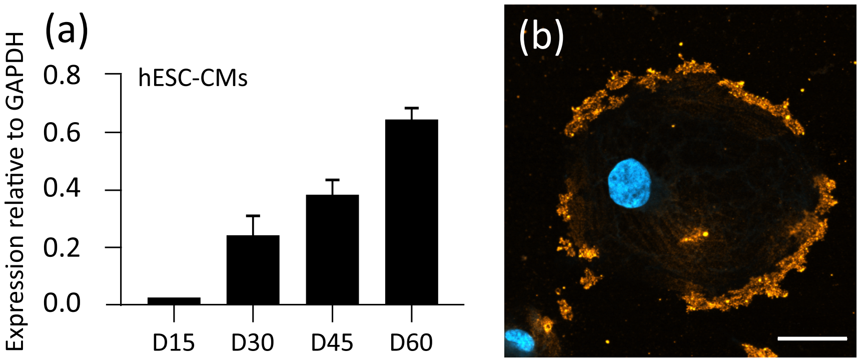

Figure S3. Expression of CD36 during hESC cardiac differentiation (A) qPCR analysis of CD36 in hESC-CMs at different days (D) of differentiation expressed as % GAPDH, n=3. (b) Confocal image of a representative hESC-CM immunostained with anti-CD36 antibody (in orange) showing cell surface expression. The nucleus (blue) was labeled with DAPI. Scale bar = 20 µm. Data shown as mean±SEM.

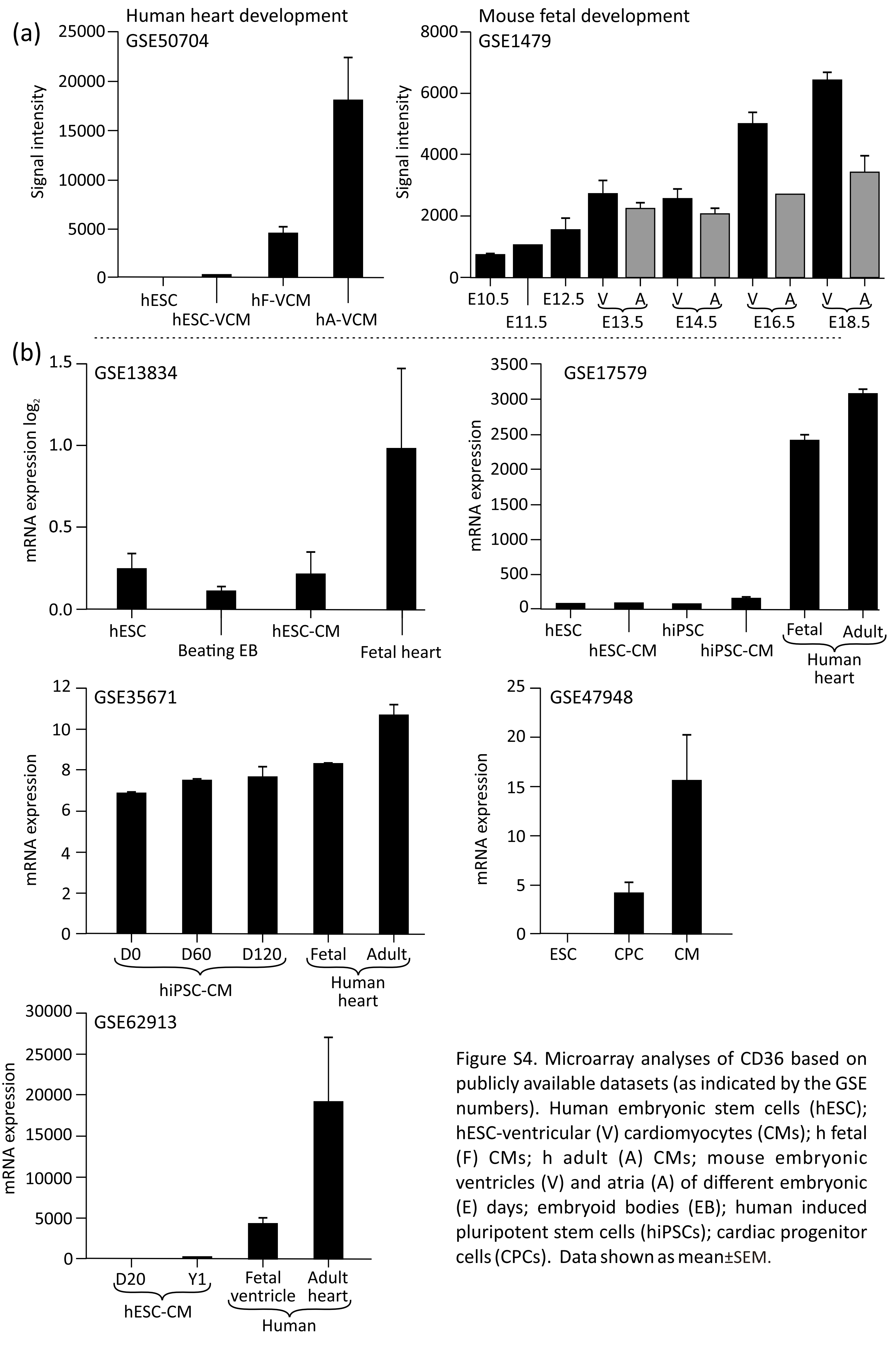

Figure S4. Microarray analyses of CD36 based on publicly available datasets (as indicated by the GSE numbers). Human embryonic stem cells (hESC); hESC-ventricular (V) cardiomyocytes (CMs); h fetal (F) CMs; h adult (A) CMs; mouse embryonic ventricles (V) and atria (A) of different embryonic (E) days; embryoid bodies (EB); human induced pluripotent stem cells (hiPSCs); cardiac progenitor cells (CPCs). Data shown as mean±SEM.

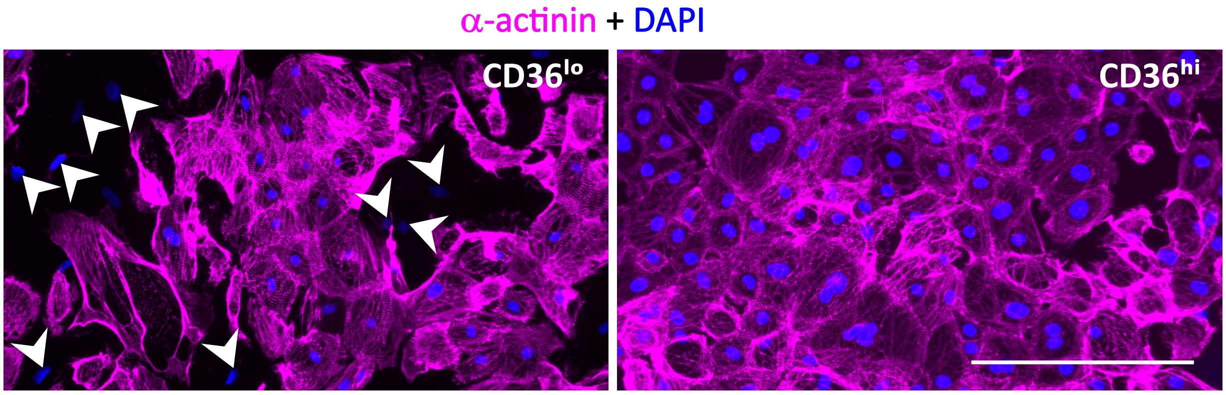

Figure S5. Sorting with anti-CD36 antibody alone enriches for α-actinin$^+$ CMs in the CD36^{hi} population. α-actinin and DAPI are in purple and blue, respectively. The arrowheads indicate non-CMs that are α-actinin$^-$, which are enriched in the CD36^{lo} cells. Scale bar = 200 µm.

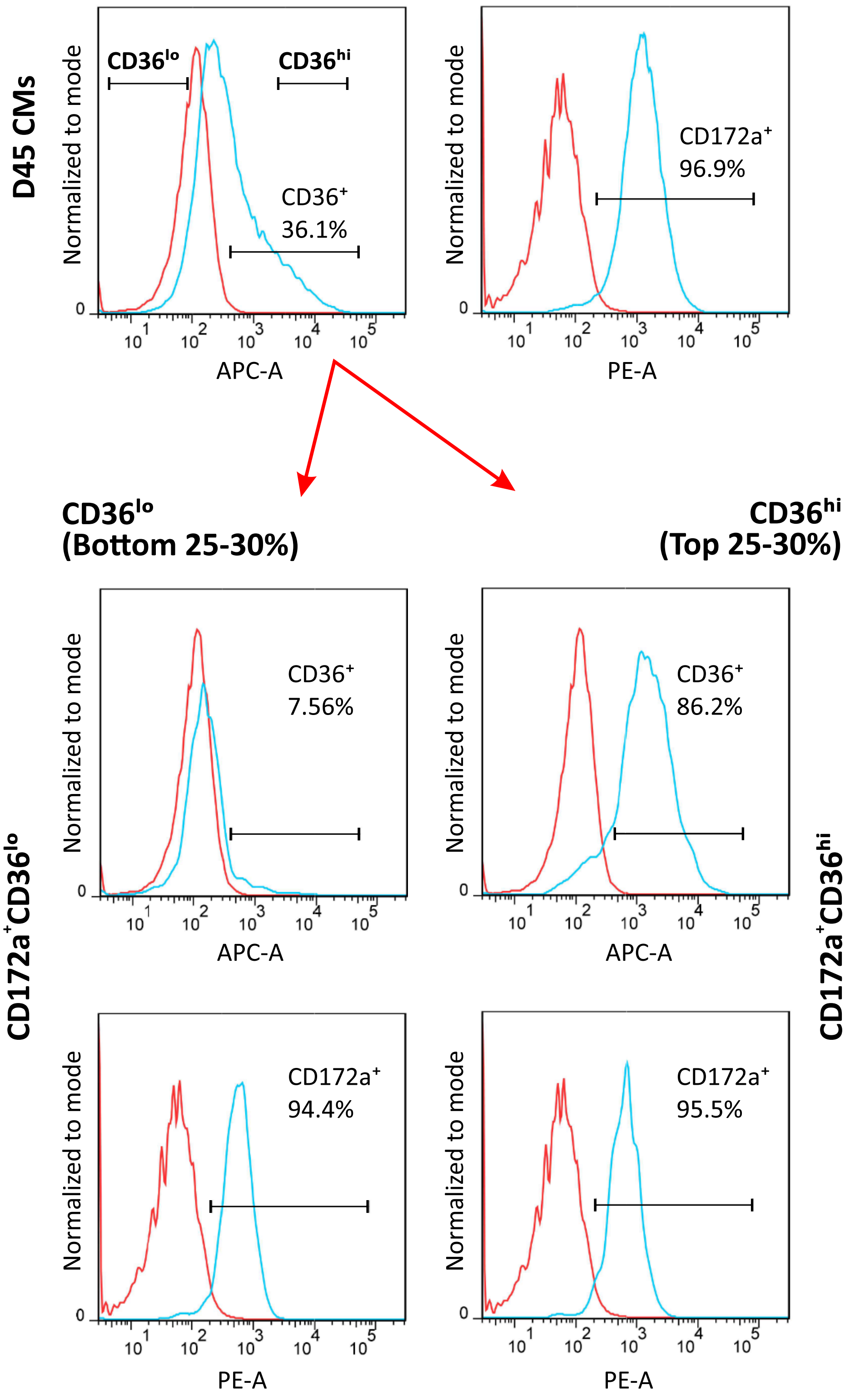

Figure S6. Sorting hESC-CMs greatly enriches the number of CMs that are CD36 positive. CD172a/SIRPA, a commonly used CM marker was included to ensure that the sorted cells consisted mainly of CMs. $CD172a^{+}CD36^{hi}$ and $CD172a^{+}CD36^{lo}$ CMs were used for subsequent experiments unless otherwise stated. Isotype controls are shown in red, whereas CD36-APC and CD172A-PE staining are shown in blue.

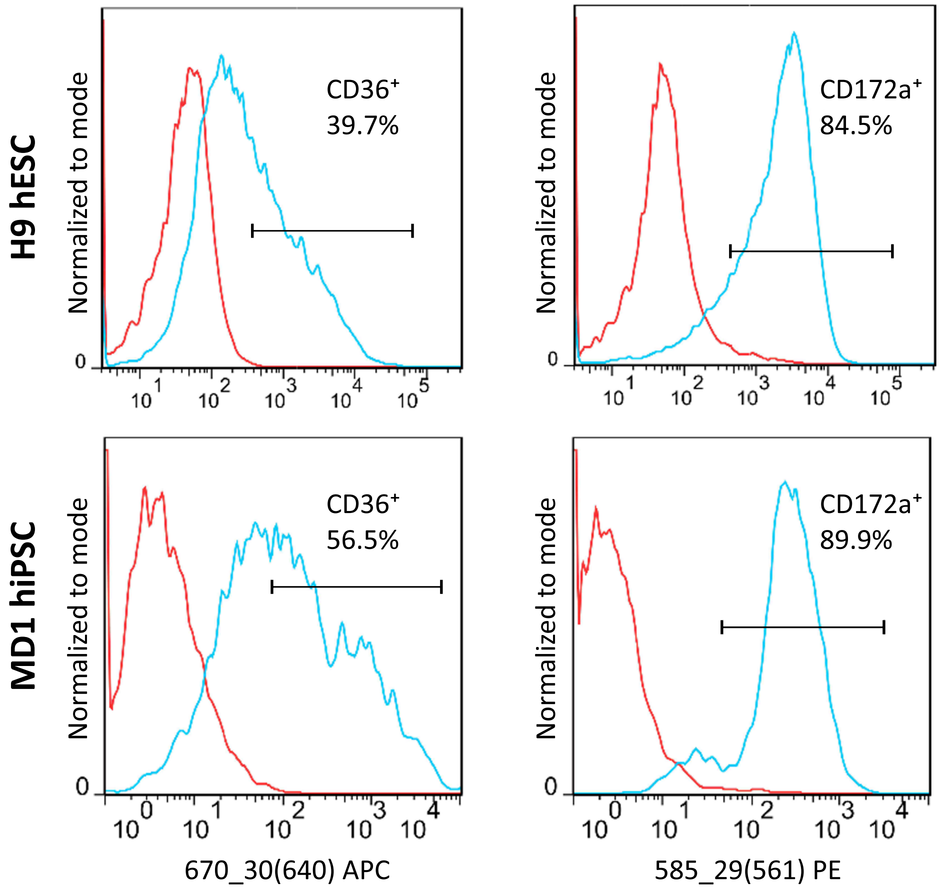

Figure S7. Expression of CD36 in hESC (H9) and hiPSC (MD1)-CMs. Flow cytometric examination of CD36 in CMs at 45±5 days of differentiation. Isotype control and CD36-APC staining are shown in red and blue, respectively.

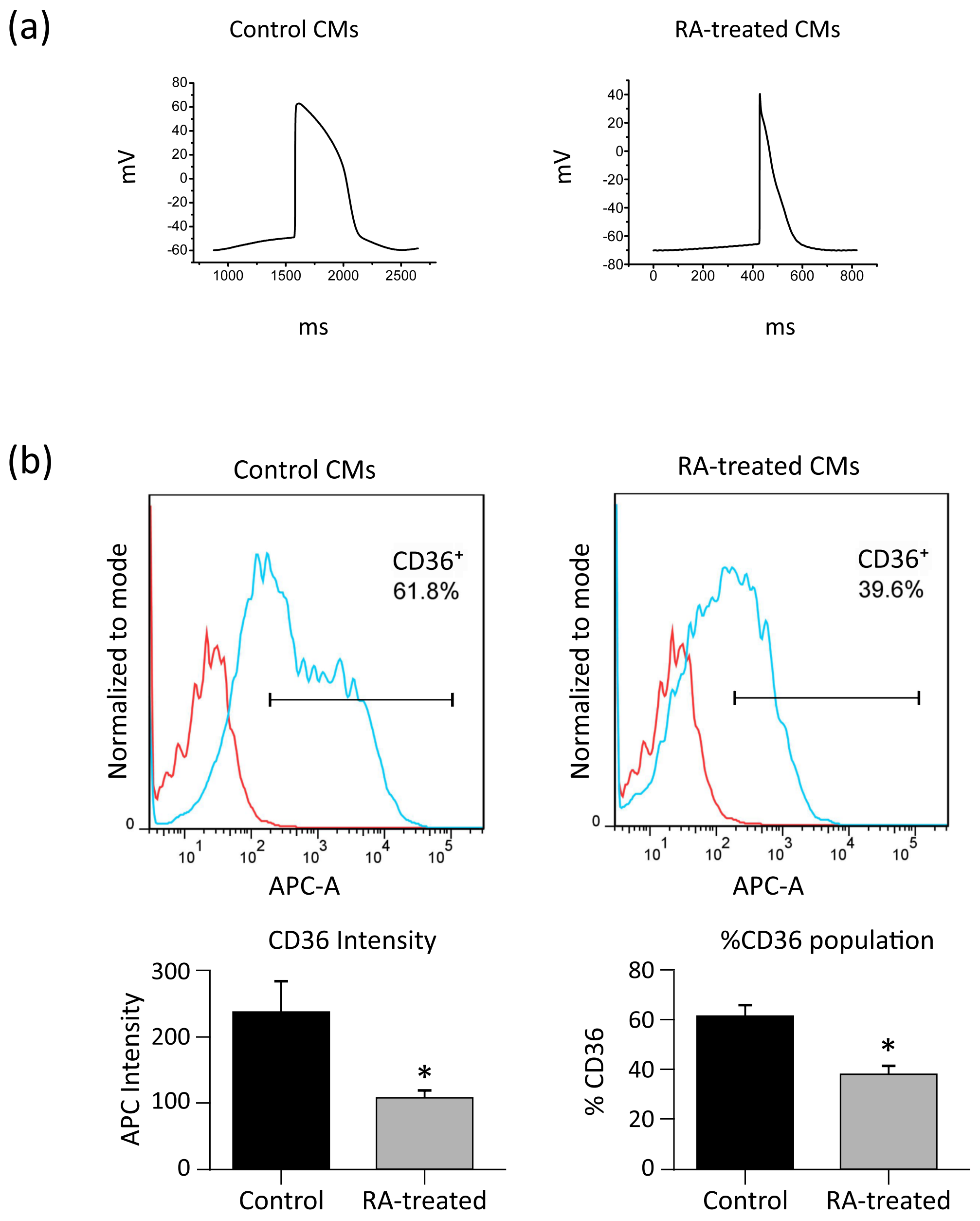

Figure S8. CD36 surface staining is higher in ventricular-like hPSC-CMs. (a) Representative action potential tracings from control and retinoic acid (RA)-treated hPSC-CMs, showing ventricular and atrial-like patterns respectively. b) CD36 staining is higher in control ventricular-like CMs than in RA-treated atrial-like CMs when analyzed by flow cytometry. * $p<0.05$. Data shown as mean±SEM.

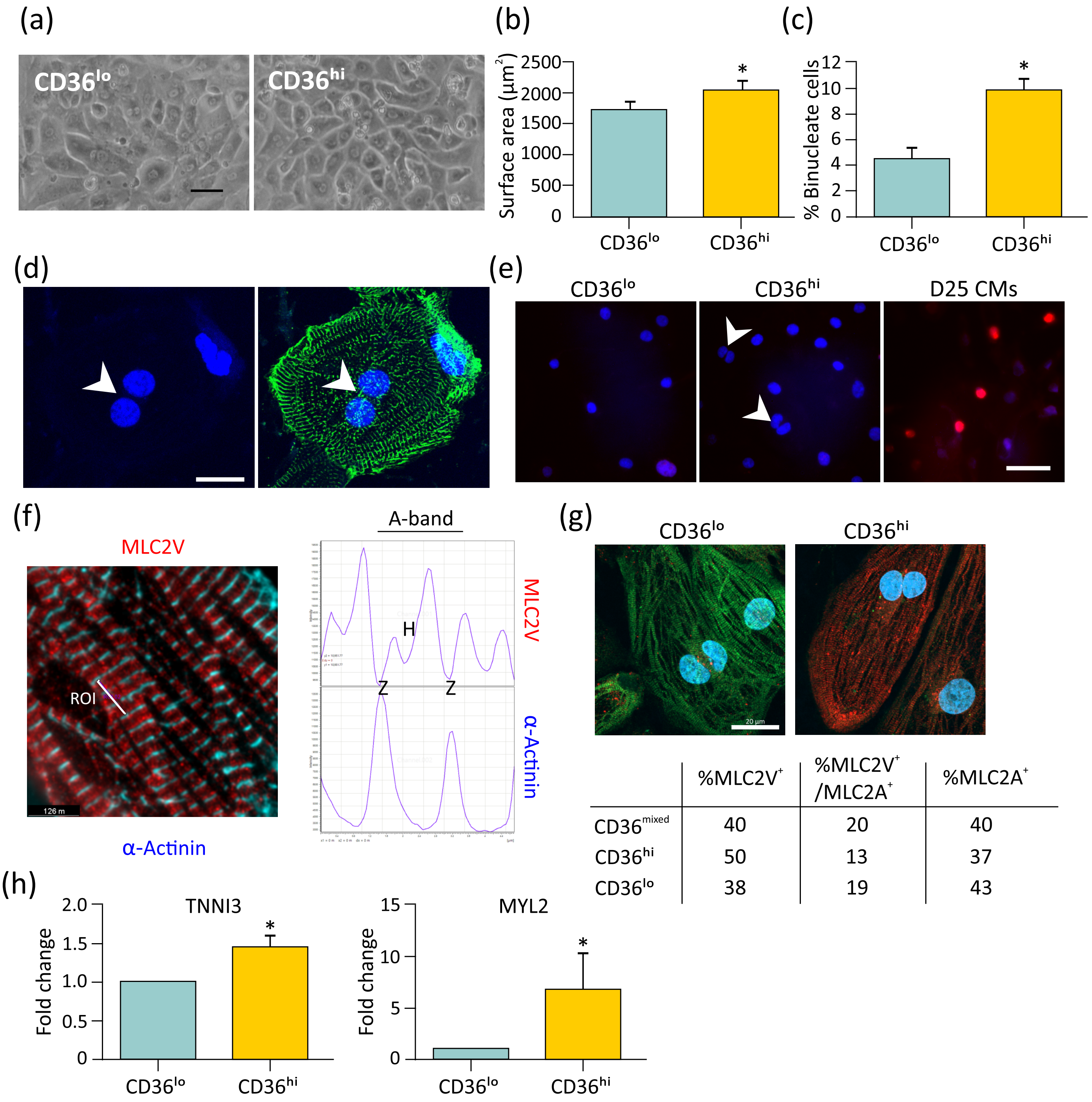

	%MLC2V+	%MLC2V+ /MLC2A+	%MLC2A+
CD36mixed	40	20	40
CD36hi	50	13	37
CD36lo	38	19	43

Figure S9. Characterisation of sorted CD36^{lo} and CD36^{hi} subpopulations. (a) Phase contrast images of sorted and replated CD36^{lo} and CD36^{hi} CMs. (b) Surface area of α-actinin^{+} cells was measured using ImageJ. CD36^{lo} (n=120), CD36^{hi} CMs (n=104). (c) % CMs with binucleated nuclei (n=5). (d) Example of a binucleate cell, which was stained with DAPI (blue) and an antibody to α-actinin (green). (e) Fluorescence images of CMs stained with anti-Ki67 antibody (red). CD36^{lo} and CD36^{hi} CMs were negative for this proliferation marker. Day 25 CMs served as positive control and contained red Ki67^{+} nuclei. DAPI labeling is in blue. In (d) and (e), the white arrowheads indicate binucleate cells. (f) Image of CD36^{hi} CM stained with anti-MLC2V (red) and α-actinin (cyan) antibodies, and line plot of region of interest (ROI) showing relative intensities. Areas corresponding to A-band (A), H-zone (H) and Z-disks (Z) are shown. (g) CD36 subpopulations were stained with anti-MLC2A (green) and MLC2V (red) antibodies. Representative image of MLC2A^{+}/MLC2V^{-} and MLC2A^{-}/MLC2V^{+} CMs prevalent in CD36^{lo} and CD36^{hi} subpopulations respectively are shown. The proportion of cells positive for MLC2V and MLC2A were determined, n=4. (h) qPCR analysis of structural genes, n=7. * $p<0.05$. Data shown as mean±SEM. Scale bars = 50 μm (in a and d) and 20 μm (in c and g).

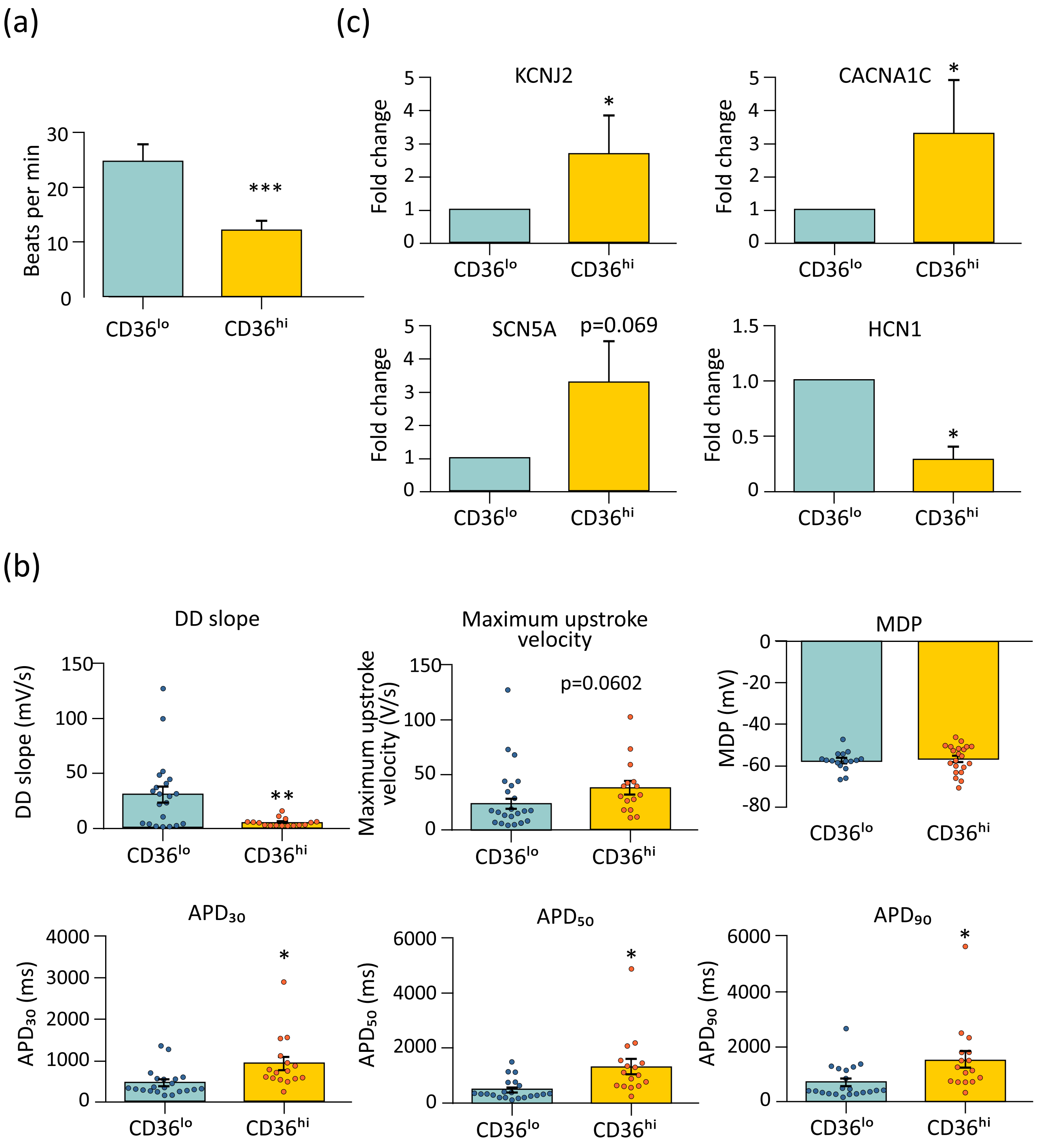

Figure S10. (a) Spontaneous beating frequency (n=7) (b) Action potential parameters of $CD36^{lo}$ (n=21) and $CD36^{hi}$ (n=16) CMs. (c) qPCR analysis of genes encoding subunits of cardiac channels. * $p \leq 0.05$, *** $p \leq 0.001$. Data shown as mean±SEM.

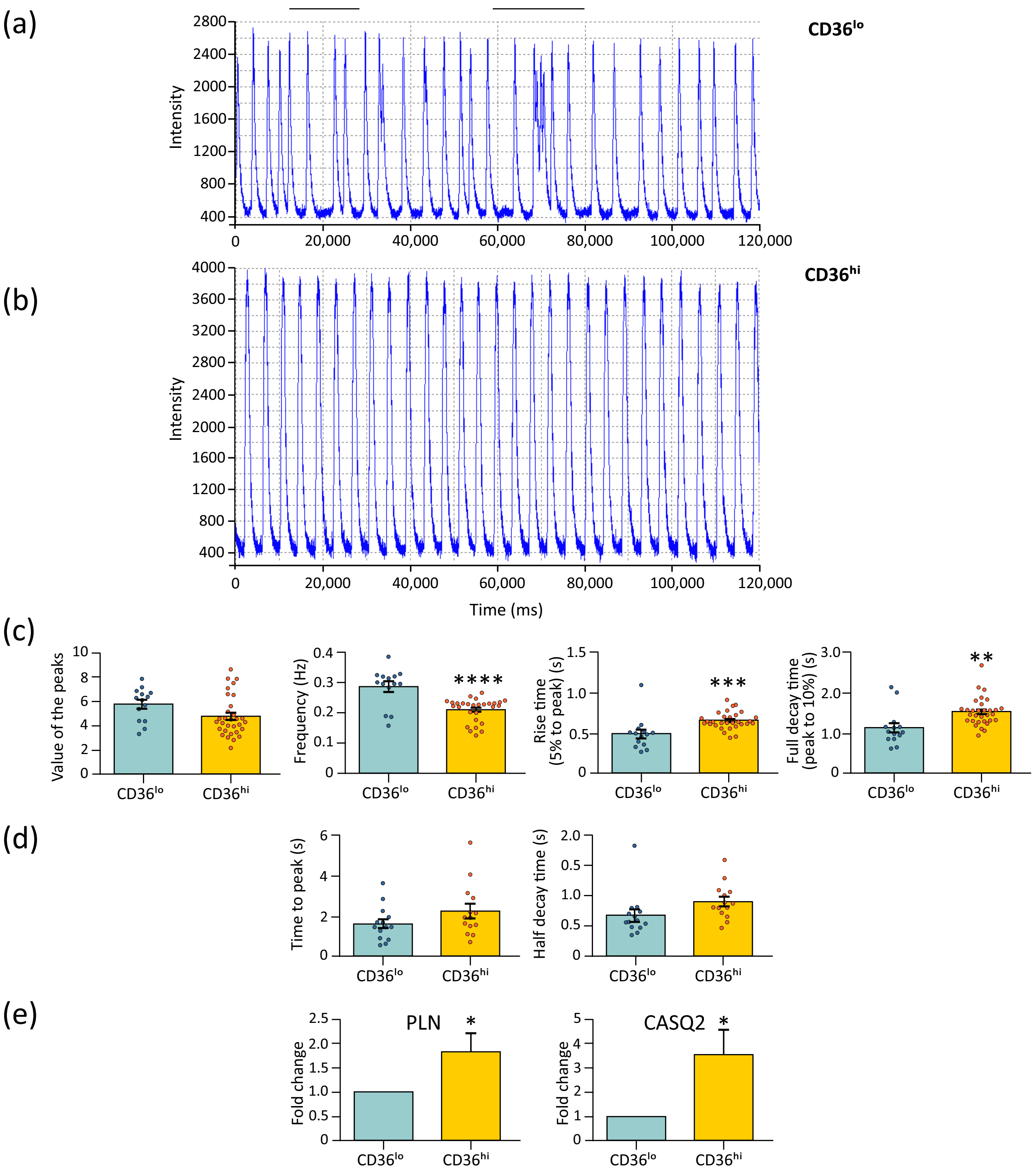

Figure S11. Representative examples and quantification of the properties of Ca^{2+} transients generated in (a) CD36^{lo} and (b) CD36^{hi} CMs. Some irregular beating could be observed in the CD36^{lo} CMs, as indicated by the black bars. Experiments were performed and confirmed in two independent laboratories, and summary data from both laboratories are shown in (c) and (d) respectively. The frequency and amplitude data for the first laboratory are presented in Fig. 1 and are omitted from (d). (e) qPCR analyses of genes important for Ca^{2+} handling. * $p \leq 0.05$, ** $p \leq 0.01$, *** $p \leq 0.001$, and **** $p \leq 0.0001$. Data shown as mean±SEM.

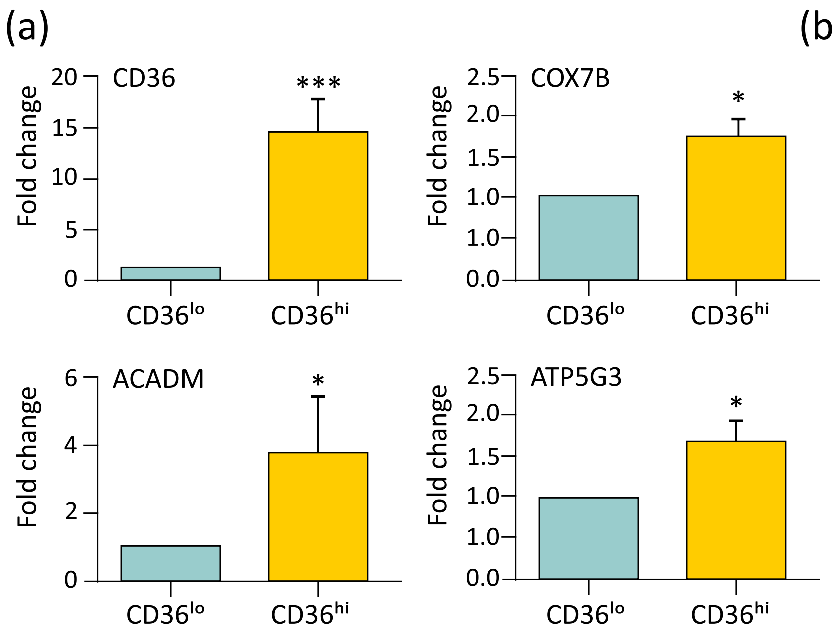

(b)

Gene symbol	P-value
PPIF	9.13E-06
COX5A	1.51E-05
UQCRFS1	1.80E-05
UQCRC1	1.12E-04
CYCS	1.86E-04
NDUFA8	5.64E-04
SDHA	6.14E-04
NDUFB9	6.75E-04
NDUFV1	6.91E-04

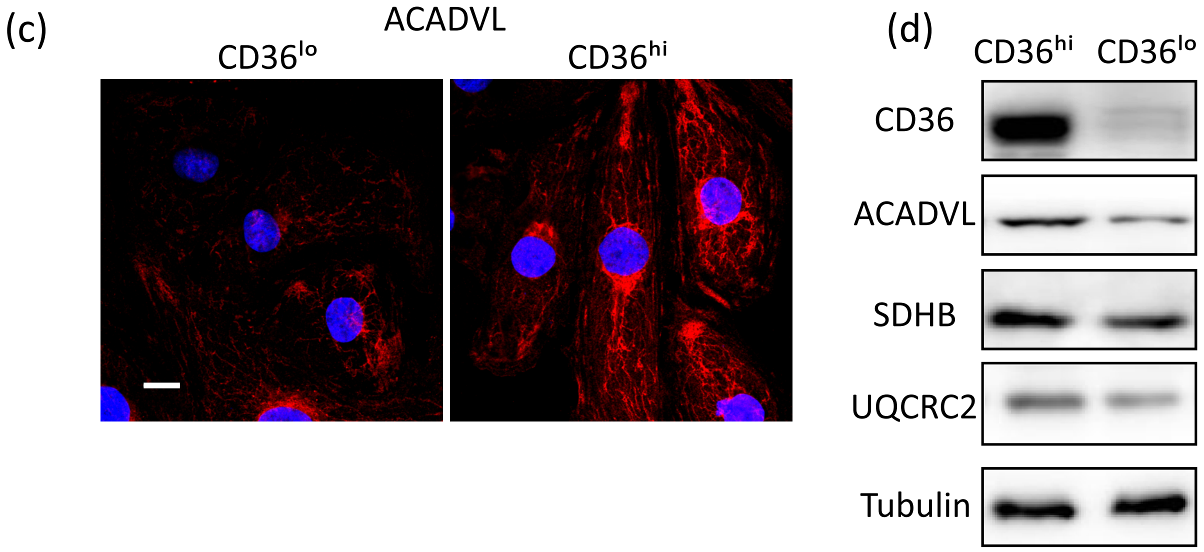

Figure S12. Analyses of CD36^{lo} and CD36^{hi} subpopulations from sorted cells. (a) qPCR analysis of selected genes in CD36^{lo} and CD36^{hi} subpopulations. RNA abundance is presented as mean ± SEM, normalised to that of GAPDH and to CD36^{lo} CMs (n=5). ACADM - Medium-chain specific acyl-CoA dehydrogenase, mitochondrial; COX7B - Cytochrome c oxidase subunit 7B, mitochondrial; ATP5G3 - ATP Synthase Membrane Subunit C Locus 3. (b) Pearson correlation between CD36 and genes involved in oxidative correlation (GO:0006119). The nine genes with most significant correlation are shown. (c) Confocal images of CD36^{lo} and CD36^{hi} hESC-CMs immunostained with anti-ACADVL (Acyl-CoA Dehydrogenase Very Long Chain) antibody (red), and counterstained with DAPI (blue). Scale bar = 10 µm. (d) Western blot showing increased protein levels of CD36, ACADVL, and succinate dehydrogenase (SDHB) in CD36^{hi} CMs, when compared with CD36^{lo} CMs. Tubulin was used as the loading control. * $p \leq 0.05$, *** $p \leq 0.001$. Data shown as mean±SEM.

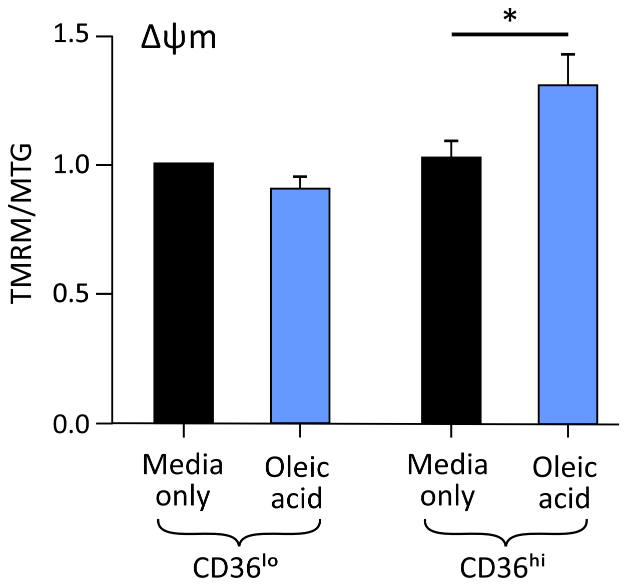

Figure S13. Fatty acid utilization measured using TMRM/MTG dyes. Fuel utilization was assessed by measuring the Δψm with/without oleic acid supplementation, normalised to CD36^{lo} CMs in media alone. Δψm was measured and calculated as TMRM/MTG, n=7. An increase in Δψm after oleic acid supplementation indicates fatty acid utilization. * $p \leq 0.05$. Data shown as mean±SEM.

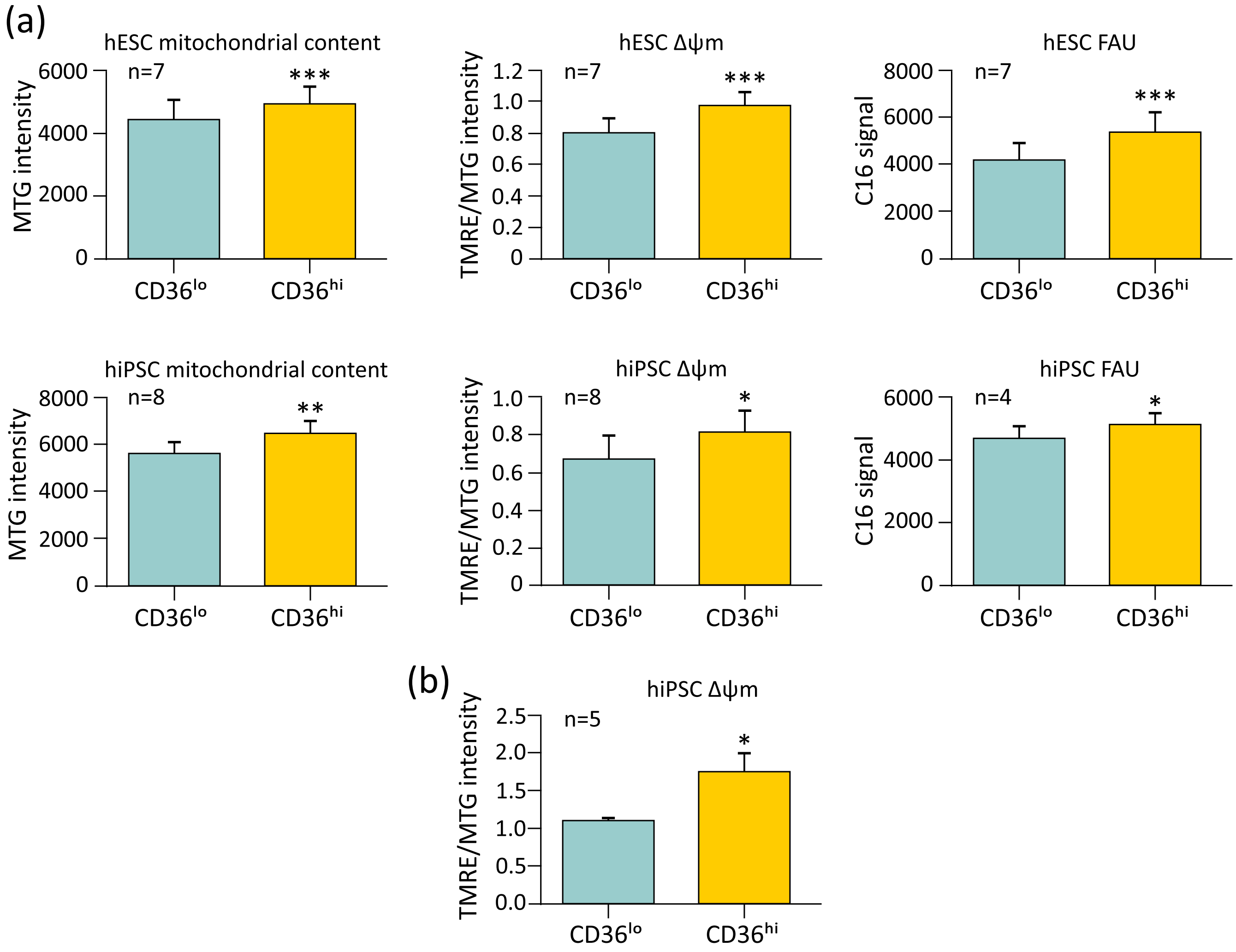

Figure S14. Mitochondrial and metabolic properties of hESC- (H7) and hiPSC- (MD1) CMs. (a) Mitochondrial content, membrane potential (Δψm) and fatty acid uptake (FAU) at D45±5 were measured by flow cytometry using MTG, TMRE/MTG and C16 palmitate fluorescent analogs, respectively. Samples were simultaneously gated for CD36 surface expression. (b) CMs were sorted on D45±5 and the Δψm of CD36^{hi} and CD36^{lo} CMs was measured 1-2 weeks after sorting using a plate-reader. * p≤ 0.05, ** p≤ 0.01, *** p≤ 0.001. Data shown as mean±SEM.

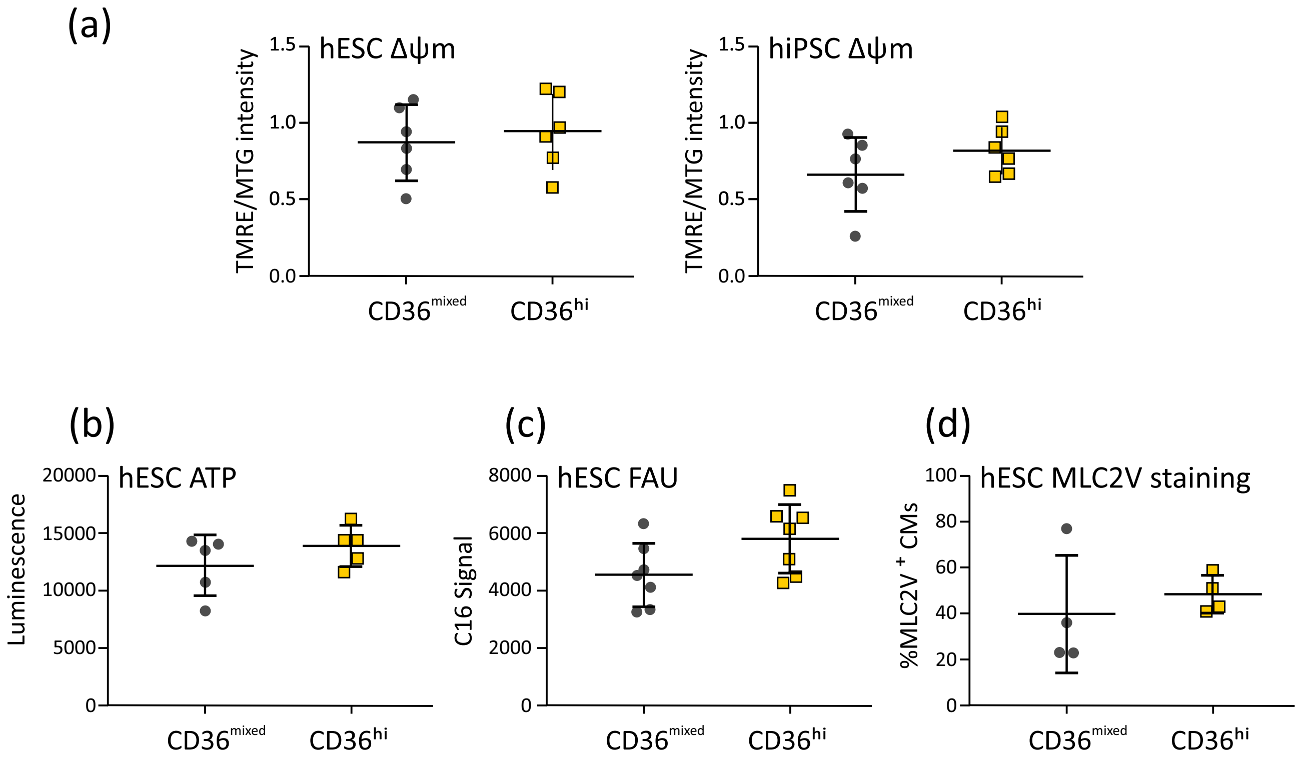

(e)

	Δψm			ATP	FAU	$\%MLC2V^+$
	hESC	hiPSC	hESC + hiPSC	hESC	hESC	hESC
$CD36^{mixed}$	28	36	33	21	17	66
$CD36^{hi}$	26	19	24	13	13	17

Figure S15. Sample variability of $CD36^{Mixed}$ and $CD36^{hi}$ CMs. (a) The Δψm was measured and calculated as TMRE/MTG in ungated CMs (mixed) and CMs gated for high CD36 expression ($CD36^{hi}$). hESC- (H7) and hiPSC- (MD1) CMs were used, n=6 for each. The same CD36 gate was applied to all the samples such that the $CD36^{hi}$ cells had similar levels of CD36 staining. (b)-(d) Comparisons were made between mixed and hESC-CMs sorted for high CD36 expression ($CD36^{hi}$). (b) ATP production was measured by luminescence, n=5. (c) The uptake of fluorescently-labeled C-16 fatty acid (FAU) was measured, n=7. (d) The proportion of cells positive for MLC2V was revealed by immunofluorescence staining, n=4. (e) The coefficient of variation (CV) was calculated among hESC- and hiPSC-CMs, n=6 for each. hESC- and hPSC-CMs were combined for analysis in the 'hESC+hiPSC' column, n=12. The next three columns show the CV of ATP, FAU and $\%MLC2V^+$ CM measurements in hESC-CMS.

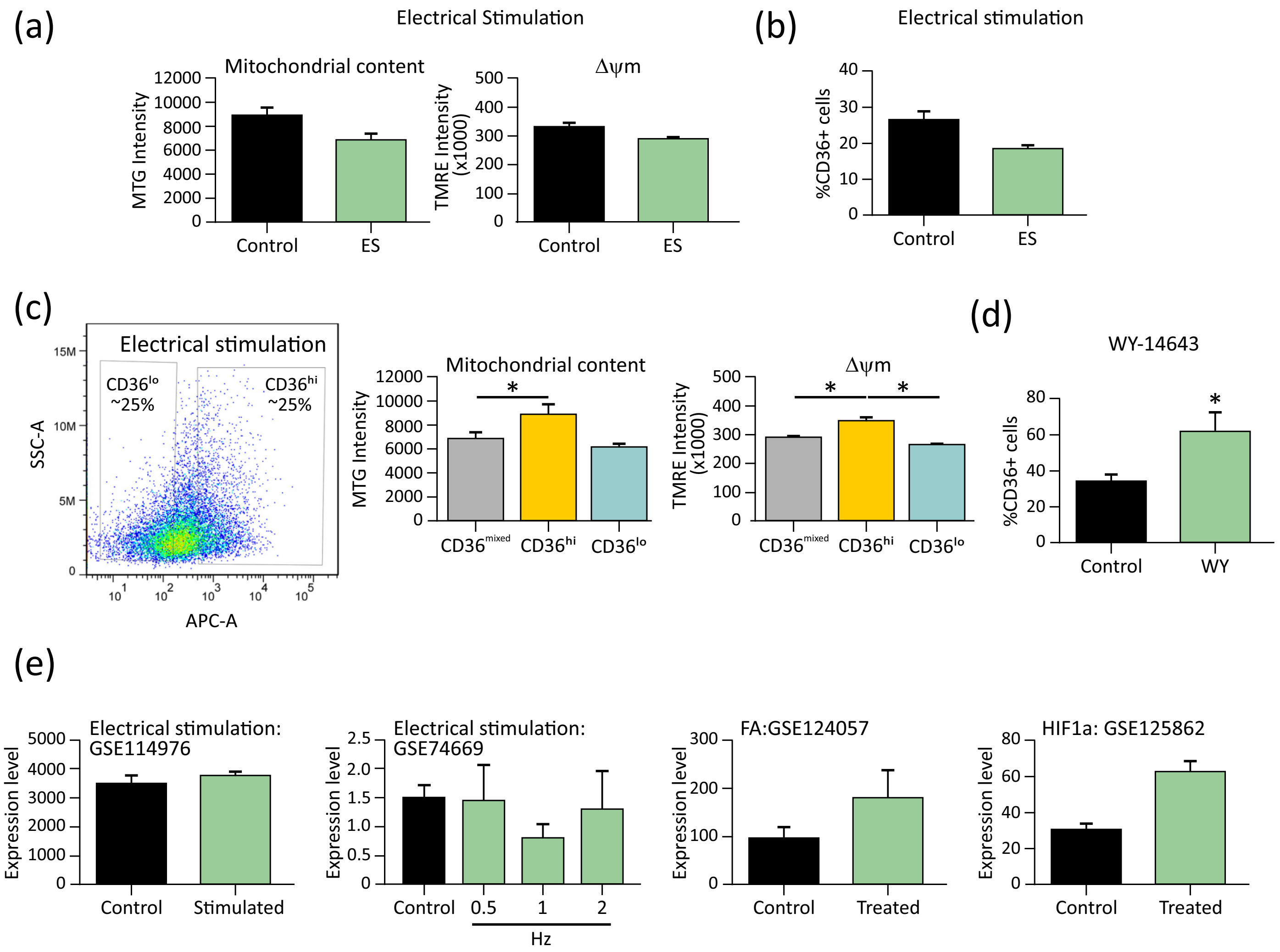

Figure S16. Correlation between CD36 expression and other protocols of maturation. (a) The effect of electrical stimulation (7 days) on mitochondrial content and Δψm were assessed using MTG and TMRE staining, followed by flow analysis (n=3). The effect of (b) electrical stimulation and (d) PPARa agonist, WY-14643 (100µM, 10 days) on CD36 were assessed by flow analysis. (c) Electrically stimulated CMs were gated for high ($CD36^{hi}$) and low ($CD36^{lo}$) CD36 expression. Mitochondrial content and Δψm of electrically stimulated cells were assessed in mixed, $CD36^{hi}$ and $CD36^{lo}$ subpopulations. (e) Transcriptomic analysis of CD36 based on publicly available datasets. * $p \leq 0.05$. Data shown as mean±SEM.

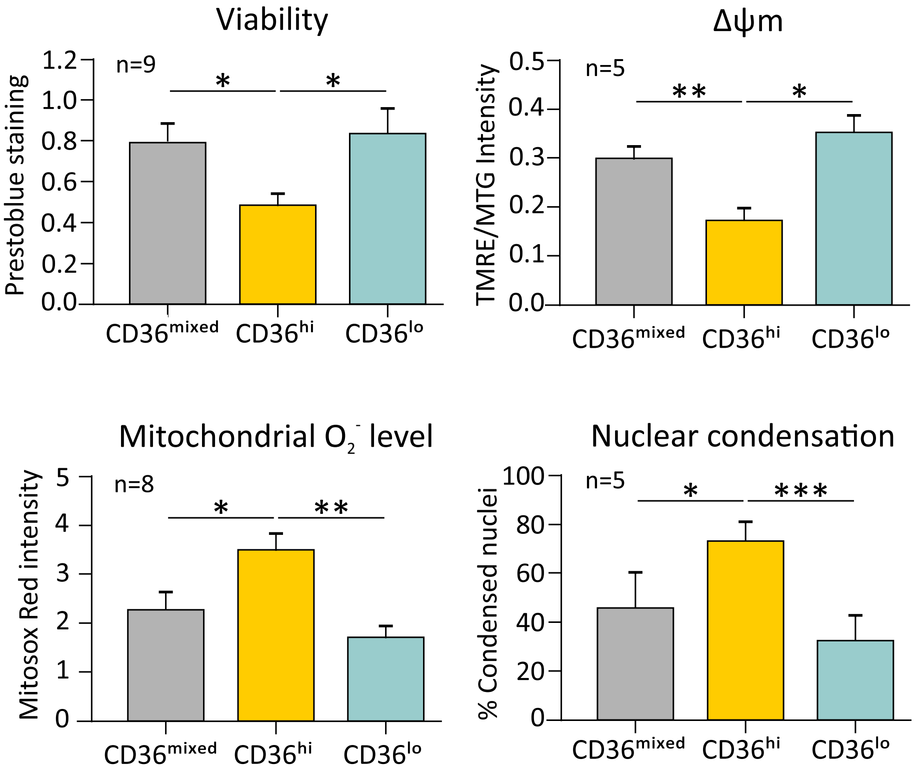

Figure S17. Effects of H_2O_2 on CD36^{mixed}, CD36^{hi} and CD36^{lo} CMs. Mitochondrial O_2^-, Δψm, viability and nuclear condensation were measured using mitosox red, TMRE/MTG, Prestoblue and Hoescht dyes, respectively. Data were normalised to untreated cells. * p≤ 0.05, ** p≤ 0.01, *** p≤ 0.001. Data shown as mean±SEM.

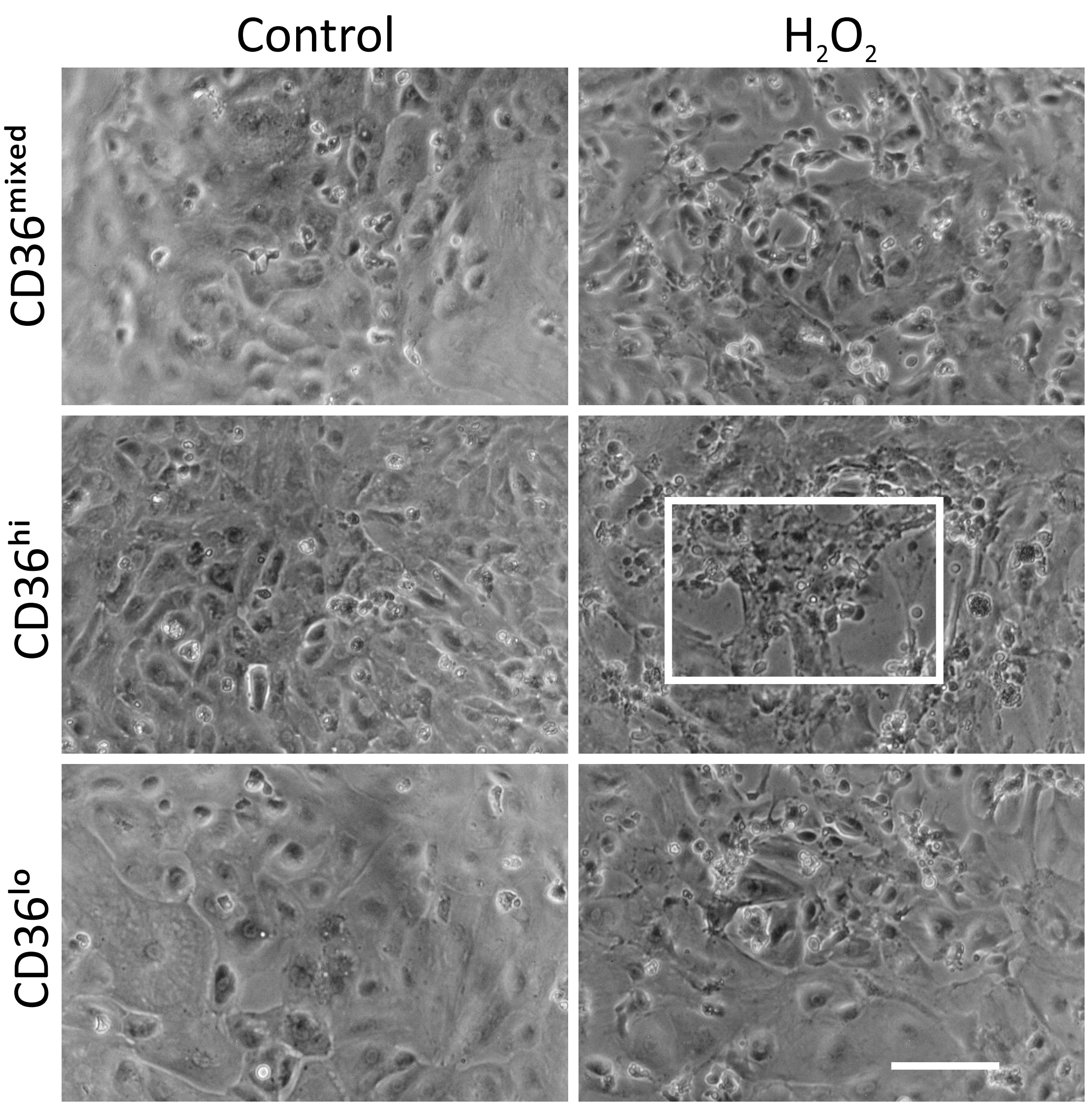

Figure S18. Morphological changes induced by H_2O_2. Phase-contrast images of $CD36^{mixed}$, $CD36^{hi}$, and $CD36^{lo}$ CMs exposed to 100 μM H_2O_2 for 30 min. $CD36^{hi}$ CMs exhibited pronounced degeneration (as shown in the white rectangle) upon treatment, whereas $CD36^{mixed}$ and $CD36^{lo}$ CMs were relatively resistant. Scale bar = 100 μm.

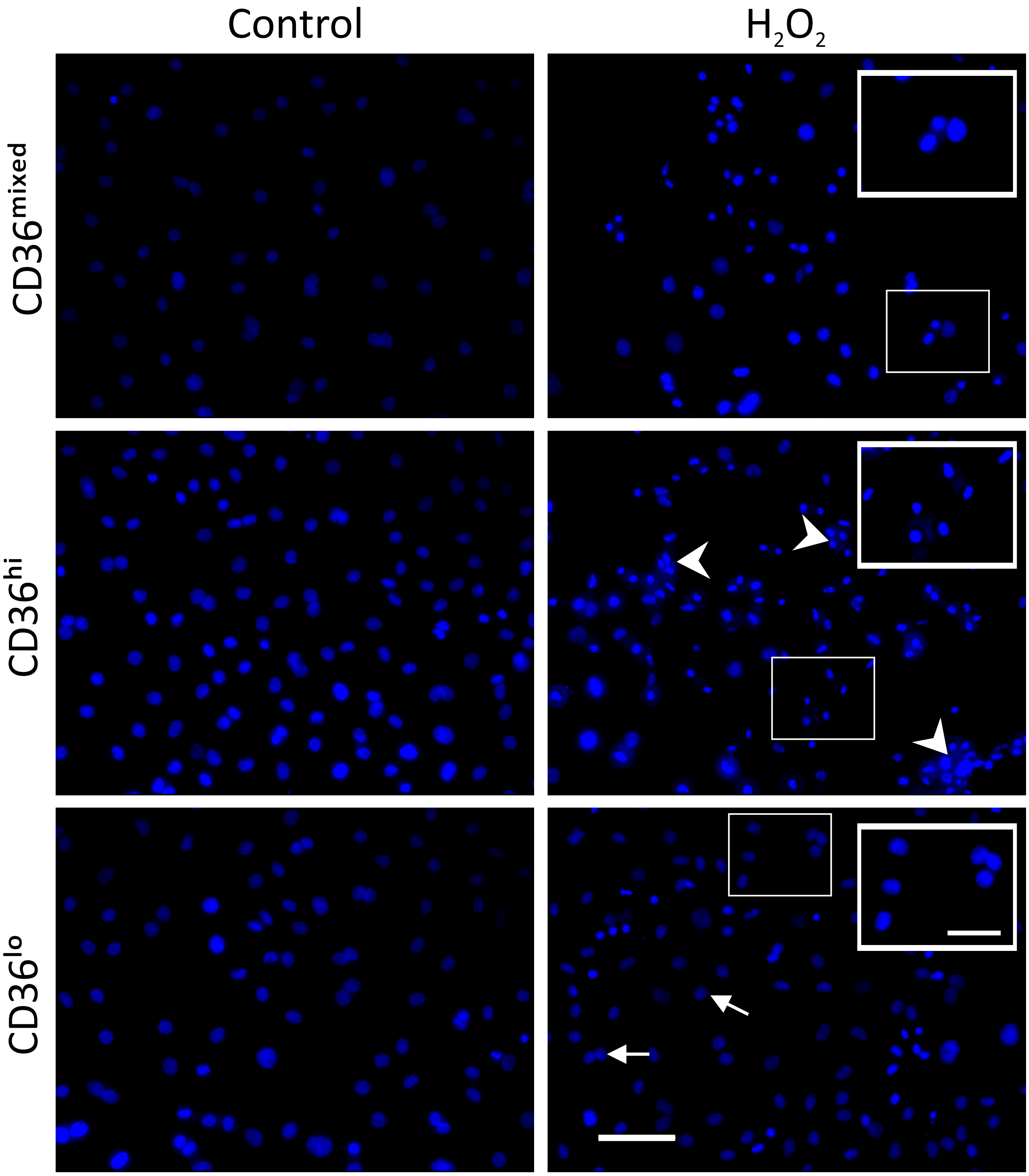

Figure S19. Nuclear condensation induced by H_2O_2. CD36^{mixed}, CD36^{hi} and CD36^{lo} CMs were exposed to 100 µM H_2O_2 for 30 min, after which the nuclei were stained with Hoescht 33342. H_2O_2-treated CD36^{hi} cultures contained a large proportion of cells with condensed, bright and irregular nuclei (see white arrowheads), whereas H_2O_2-treated CD36^{lo} CMs mostly had 'normal' nuclei that were bigger and more regular (see white arrows). The panel insets, show magnified views of selected areas. Scale bars = 50 µm (main images) and 25 µm (inset images).

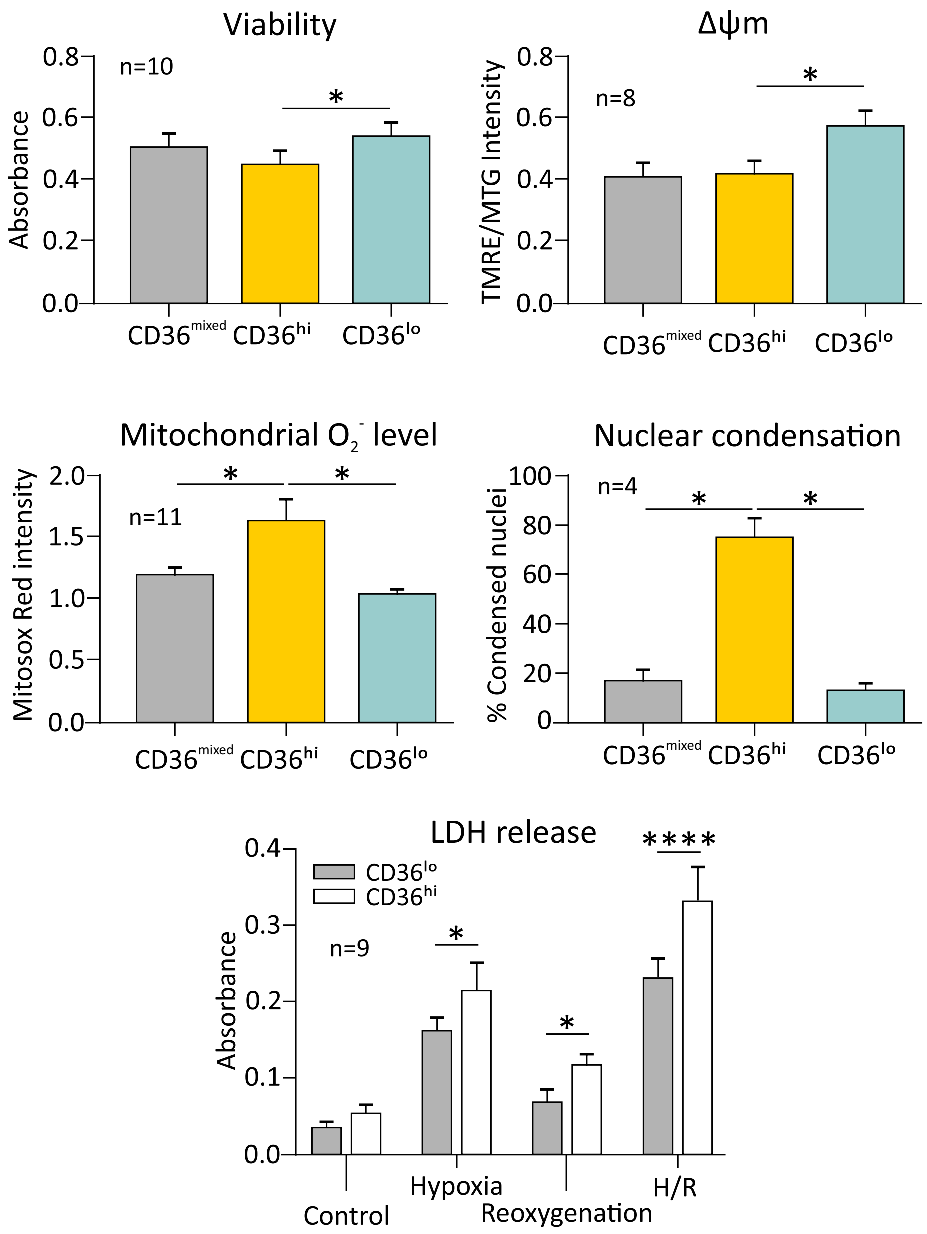

Figure S20. Effects of hypoxia/reoxygenation on CD36^{mixed}, CD36^{hi} and CD36^{lo} CMs. Mitochondrial O_2, Δψm, viability and nuclear condensation were measured using mitosox red, TMRE/MTG, MTT and Hoescht dyes, respectively. Data were normalised to untreated cells. LDH was measured after hypoxia/reoxygenation treatment of the same cells. H/R is the composite of these two measurements. * $p \leq 0.05$, * *** $p \leq 0.0001$. Data shown as mean±SEM.

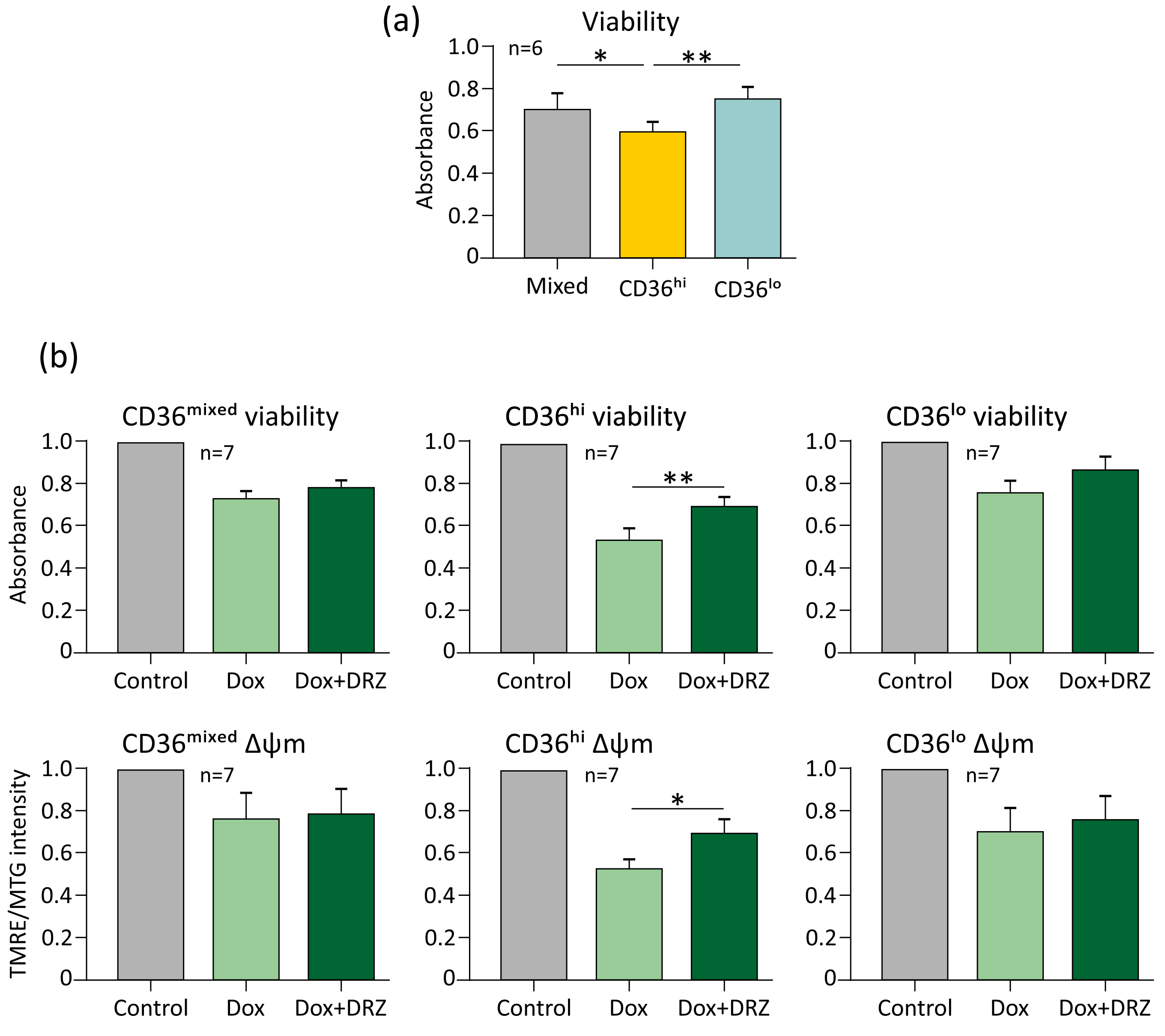

Figure S21. Effect of Dox on CD36^{mixed}, CD36^{hi} and CD36^{lo} CMs derived from hiPSCs and hESCs.
(a) CD36^{mixed}, CD36^{hi} and CD36^{lo} CMs derived from hiPSC (MD1) were treated with doxorubicin (Dox; 1 µM) for 24 h. (b) CD36^{mixed}, CD36^{hi} and CD36^{lo} CMs derived from hESC (H7) were pre-treated with dexrazoxane (DRZ, 200 µM) for one hour prior to co-treatment with doxorubicin (Dox; 1 µM) for 24 h. Viability was measured using the XTT assay, and the $\Delta\psi m$ was measured using TMRE and MTG dyes. *$p<0.05$, **$p<0.01$. Data shown as mean±SEM.

Gene Symbol	# cell types /lines	Gene Symbol	# cell types /lines	Gene Symbol	# cell types /lines
SIRPB1	9	ITGA3	37	BST2	34
NRP1	27	BCAM	25	PROCR	26
PROM1	7	CD276	36	PDGFRA	8
TFRC	39	ENTPD1	15	INSR	29
ITGB1	45	ACE	12	PRNP	45
ITGAV	42	JAG1	18	ITGB3	9
IGF1R	38	CD47	43	LAMP2	36
ITGA5	38	CD37	13	CD24	0
LAMP1	39	CD300C	0	ITGA2	30
NCAM1	29	MST1R	14	CD55	39
CD59	45	CD28	5	SEMA4D	26
CDH2	26	CD46	37	PLXNC1	17
CD58	40	CD44	43	CD200	19
VCAM1	11	MCAM	25	IGSF8	36
NT5E	30	CD36	6	CD82	29
BSG	47	PTPRJ	40	SEMA7A	21
KDR	0	FGFR1	16	MME	30
ATP1B3	45	ADAM10	43	CD7	7
ITGA1	32	TNFRSF10C	0		
LRP1	28	ICOSLG	21		
ALCAM	41	ICAM1	0		
CD109	36	CD63	42		
PTGFRN	30	F3	30		
MRC2	31	PVR	27		
ERBB2	14	IFNGR1	28		
GGT1	23	ITGA6	31		
LIFR	11	FAS	26		
SIRPA	31	IL6ST	27		
PECAM1	14	ADAM17	26		

Table S1. Tissue specificity of CD molecules detected in the CSC experiment. The presence of 77 CD molecules among 47 different human cell types/lines was determined by comparison with the CSPA database. # denotes the number of cell types/lines, which express each CD molecule. CD molecules that were detected in ≤20% (9/47) of cell types/lines are highlighted in red.

Table S2A Gene Ontology analysis of genes up-regulated in CD36hi CMs. FDR<5%

Category	Term	PValue	FDR
GOTERM_CC_DIRECT	GO:0005743~mitochondrial inner membrane	2.46E-17	3.21E-14
GOTERM_CC_DIRECT	GO:0005739~mitochondrion	3.20E-15	4.20E-12
GOTERM_BP_DIRECT	GO:0006123~mitochondrial electron transpo	8.11E-11	1.30E-07
GOTERM_BP_DIRECT	GO:1902600~hydrogen ion transmembrane t	1.20E-06	0.001924
GOTERM_BP_DIRECT	GO:0007062~sister chromatid cohesion	8.04E-06	0.012919
GOTERM_CC_DIRECT	GO:0005751~mitochondrial respiratory chain	1.67E-05	0.021815
GOTERM_CC_DIRECT	GO:0005753~mitochondrial proton-transport	1.29E-04	0.168432
GOTERM_BP_DIRECT	GO:0042776~mitochondrial ATP synthesis co	1.42E-04	0.227094
GOTERM_BP_DIRECT	GO:0019915~lipid storage	2.44E-04	0.39071
GOTERM_BP_DIRECT	GO:0007059~chromosome segregation	2.56E-04	0.411056
GOTERM_BP_DIRECT	GO:0000070~mitotic sister chromatid segreg	2.87E-04	0.460252
GOTERM_CC_DIRECT	GO:0005747~mitochondrial respiratory chain	3.87E-04	0.503665
GOTERM_BP_DIRECT	GO:0006120~mitochondrial electron transpo	4.31E-04	0.68999
GOTERM_CC_DIRECT	GO:0005759~mitochondrial matrix	0.001033	1.339083
GOTERM_BP_DIRECT	GO:0006281~DNA repair	0.001058	1.686268
GOTERM_BP_DIRECT	GO:0007067~mitotic nuclear division	0.001597	2.534502
GOTERM_BP_DIRECT	GO:0051301~cell division	0.002215	3.499522

Table S2B Gene Ontology analysis of genes down-regulated in CD36hi CMs. FDR<5%

Category	Term	PValue	FDR
GOTERM_BP_DIRECT	GO:0007155~cell adhesion	5.79E-18	1.03E-14
GOTERM_BP_DIRECT	GO:0030198~extracellular matrix organizatio	8.03E-17	2.00E-13
GOTERM_CC_DIRECT	GO:0005886~plasma membrane	1.35E-14	1.84E-11
GOTERM_CC_DIRECT	GO:0005578~proteinaceous extracellular ma	7.73E-13	1.05E-09
GOTERM_CC_DIRECT	GO:0005887~integral component of plasma r	8.89E-12	1.21E-08
GOTERM_CC_DIRECT	GO:0005615~extracellular space	1.13E-11	1.53E-08
GOTERM_CC_DIRECT	GO:0031012~extracellular matrix	1.22E-11	1.66E-08
GOTERM_BP_DIRECT	GO:0007411~axon guidance	1.75E-11	3.11E-08
GOTERM_BP_DIRECT	GO:0007399~nervous system development	6.36E-10	1.13E-06
GOTERM_CC_DIRECT	GO:0005576~extracellular region	1.12E-09	1.52E-06
GOTERM_CC_DIRECT	GO:0005925~focal adhesion	4.63E-09	6.31E-06
GOTERM_CC_DIRECT	GO:0009986~cell surface	7.08E-09	9.64E-06
GOTERM_CC_DIRECT	GO:0043005~neuron projection	2.16E-07	2.94E-04
GOTERM_CC_DIRECT	GO:0030424~axon	2.76E-07	3.76E-04
GOTERM_CC_DIRECT	GO:0014069~postsynaptic density	9.60E-07	0.001308
GOTERM_BP_DIRECT	GO:0030199~collagen fibril organization	1.02E-06	0.001804
GOTERM_BP_DIRECT	GO:0030335~positive regulation of cell migra	1.10E-06	0.001957
GOTERM_BP_DIRECT	GO:0007160~cell-matrix adhesion	1.33E-06	0.002357
GOTERM_BP_DIRECT	GO:0030574~collagen catabolic process	1.49E-06	0.002652
GOTERM_BP_DIRECT	GO:0001501~skeletal system development	1.54E-06	0.002727
GOTERM_BP_DIRECT	GO:0007165~signal transduction	3.31E-06	0.005883
GOTERM_BP_DIRECT	GO:0050919~negative chemotaxis	3.50E-06	0.006225
GOTERM_BP_DIRECT	GO:0007229~integrin-mediated signaling pat	3.97E-06	0.007052
GOTERM_BP_DIRECT	GO:0001666~response to hypoxia	7.30E-06	0.012972
GOTERM_CC_DIRECT	GO:0030054~cell junction	3.77E-05	0.051295
GOTERM_BP_DIRECT	GO:0071526~semaphorin-plexin signaling pat	3.00E-05	0.053367

GOTERM_CC_DIRECT	GO:0005794~Golgi apparatus	4.03E-05	0.054911
GOTERM_BP_DIRECT	GO:0001755~neural crest cell migration	3.17E-05	0.056335
GOTERM_BP_DIRECT	GO:0051965~positive regulation of synapse a	5.48E-05	0.097368
GOTERM_CC_DIRECT	GO:0008305~integrin complex	8.10E-05	0.110236
GOTERM_CC_DIRECT	GO:0005788~endoplasmic reticulum lumen	9.70E-05	0.13201
GOTERM_BP_DIRECT	GO:0090184~positive regulation of kidney de	8.64E-05	0.153447
GOTERM_BP_DIRECT	GO:0007156~homophilic cell adhesion via pla	1.51E-04	0.268702
GOTERM_CC_DIRECT	GO:0070062~extracellular exosome	2.24E-04	0.30467
GOTERM_BP_DIRECT	GO:0051764~actin crosslink formation	1.74E-04	0.308362
GOTERM_BP_DIRECT	GO:0010976~positive regulation of neuron pr	1.95E-04	0.346509
GOTERM_BP_DIRECT	GO:0010977~negative regulation of neuron p	2.06E-04	0.366067
GOTERM_CC_DIRECT	GO:0045211~postsynaptic membrane	2.86E-04	0.388318
GOTERM_BP_DIRECT	GO:0048846~axon extension involved in axor	2.55E-04	0.451986
GOTERM_BP_DIRECT	GO:0048661~positive regulation of smooth m	2.60E-04	0.461769
GOTERM_BP_DIRECT	GO:0008284~positive regulation of cell prolif	3.31E-04	0.585839
GOTERM_BP_DIRECT	GO:0014032~neural crest cell development	3.60E-04	0.637854
GOTERM_BP_DIRECT	GO:0009611~response to wounding	3.66E-04	0.648894
GOTERM_BP_DIRECT	GO:0001503~ossification	4.01E-04	0.710588
GOTERM_CC_DIRECT	GO:0043025~neuronal cell body	5.52E-04	0.74925
GOTERM_BP_DIRECT	GO:0043410~positive regulation of MAPK cas	4.40E-04	0.77965
GOTERM_BP_DIRECT	GO:0007157~heterophilic cell-cell adhesion v	4.65E-04	0.823583
GOTERM_CC_DIRECT	GO:0016021~integral component of membra	6.27E-04	0.85016
GOTERM_CC_DIRECT	GO:0045121~membrane raft	6.73E-04	0.913148
GOTERM_BP_DIRECT	GO:0016337~single organismal cell-cell adhes	5.47E-04	0.968009
GOTERM_CC_DIRECT	GO:0043204~perikaryon	7.44E-04	1.007948
GOTERM_BP_DIRECT	GO:0001525~angiogenesis	5.89E-04	1.04063
GOTERM_BP_DIRECT	GO:0008360~regulation of cell shape	5.97E-04	1.054941
GOTERM_BP_DIRECT	GO:0010811~positive regulation of cell-subst	6.14E-04	1.085827
GOTERM_BP_DIRECT	GO:0007507~heart development	6.82E-04	1.203931
GOTERM_BP_DIRECT	GO:0001938~positive regulation of endotheli	6.83E-04	1.206641
GOTERM_BP_DIRECT	GO:0048843~negative regulation of axon ext	6.93E-04	1.224224
GOTERM_BP_DIRECT	GO:0001764~neuron migration	7.44E-04	1.313733
GOTERM_BP_DIRECT	GO:0071300~cellular response to retinoic aci	7.53E-04	1.328709
GOTERM_CC_DIRECT	GO:0005581~collagen trimer	0.001044	1.41247
GOTERM_CC_DIRECT	GO:0005911~cell-cell junction	0.001073	1.450876
GOTERM_BP_DIRECT	GO:0042476~odontogenesis	8.31E-04	1.466415
GOTERM_BP_DIRECT	GO:0035987~endodermal cell differentiation	8.31E-04	1.466415
GOTERM_BP_DIRECT	GO:0048566~embryonic digestive tract devel	8.57E-04	1.512056
GOTERM_BP_DIRECT	GO:0001934~positive regulation of protein pl	9.15E-04	1.613461
GOTERM_BP_DIRECT	GO:0040007~growth	9.89E-04	1.742121
GOTERM_BP_DIRECT	GO:0032355~response to estradiol	0.001034	1.821278
GOTERM_BP_DIRECT	GO:0007612~learning	0.001041	1.832704
GOTERM_BP_DIRECT	GO:0030334~regulation of cell migration	0.00109	1.918691
GOTERM_BP_DIRECT	GO:0006954~inflammatory response	0.001125	1.980431
GOTERM_BP_DIRECT	GO:0060021~palate development	0.001299	2.282329
GOTERM_BP_DIRECT	GO:0051017~actin filament bundle assembly	0.001369	2.404446
GOTERM_BP_DIRECT	GO:0036120~cellular response to platelet-de	0.001378	2.420832
GOTERM_BP_DIRECT	GO:0007219~Notch signaling pathway	0.0015	2.631749
GOTERM_BP_DIRECT	GO:0007494~midgut development	0.001634	2.862664
GOTERM_BP_DIRECT	GO:0021675~nerve development	0.001634	2.862664

GOTERM_BP_DIRECT	GO:0016525~negative regulation of angioger	0.001717	3.006047
GOTERM_BP_DIRECT	GO:0048666~neuron development	0.001725	3.020873
GOTERM_BP_DIRECT	GO:0003007~heart morphogenesis	0.001847	3.231712
GOTERM_BP_DIRECT	GO:0071230~cellular response to amino acid	0.001931	3.376395
GOTERM_BP_DIRECT	GO:0007166~cell surface receptor signaling p	0.001998	3.490426
GOTERM_BP_DIRECT	GO:0014911~positive regulation of smooth m	0.002088	3.644543
GOTERM_BP_DIRECT	GO:0010718~positive regulation of epithelial	0.002128	3.71341
GOTERM_CC_DIRECT	GO:0043235~receptor complex	0.002916	3.898072
GOTERM_BP_DIRECT	GO:0060326~cell chemotaxis	0.002261	3.941923
GOTERM_BP_DIRECT	GO:0072015~glomerular visceral epithelial ce	0.002285	3.982834
GOTERM_BP_DIRECT	GO:0060221~retinal rod cell differentiation	0.002321	4.044558
GOTERM_BP_DIRECT	GO:0043588~skin development	0.002438	4.243638
GOTERM_BP_DIRECT	GO:0001701~in utero embryonic developmei	0.002472	4.301033
GOTERM_BP_DIRECT	GO:0042493~response to drug	0.002483	4.319759
GOTERM_CC_DIRECT	GO:0016324~apical plasma membrane	0.003363	4.484197
GOTERM_BP_DIRECT	GO:0001649~osteoblast differentiation	0.002634	4.577171
GOTERM_BP_DIRECT	GO:0001822~kidney development	0.002866	4.97125
GOTERM_CC_DIRECT	GO:0001725~stress fiber	0.003752	4.99055

Table S3A Genes up-regulated in CD36hi CMs, $p<0.05$
Fold Change is calculated as CD36hi/CD36lo CMs, >1.4

Gene Symbol	Fold change
CD36	8.3
PMEL	5.1
ASB15	4.3
ATP1A3	4.2
CRB1	3.9
NMRK2	3.3
TRH	2.9
SCTR	2.9
LINC01505	2.7
CKM	2.7
C11orf21	2.6
GLP1R	2.6
APOE	2.5
N4BP3	2.5
CHRFAM7A	2.4
ST8SIA5	2.4
ARL11	2.3
RGS16	2.2
MAP3K15	2.2
RAB26	2.2
COX6A2	2.1
UAP1L1	2.1
SLC44A3	2.1
CAPS2	2.1
UGT3A2	2.1
PAPLN	2.1
LURAP1	2.1
SHISA3	2.0
SNHG4	2.0
KIAA0040	2.0
SHBG	2.0
ABCC9	2.0
NPL	2.0
ANKRD34C	2.0
REEP6	2.0
ARHGAP27	2.0
MAOB	2.0
BSPRY	2.0
MDGA2	2.0
FANCB	1.9
ZDHHC23	1.9
KCTD19	1.9
C1QTNF1	1.9

C2orf72	1.9
ANKRD9	1.9
LINGO1	1.9
ITPKA	1.9
LMOD3	1.9
GIPR	1.9
CLIC4P3	1.9
CHRNA7	1.9
CCDC54	1.9
SEC14L5	1.9
LDHD	1.9
SYNGR3	1.9
HEY2	1.9
TNFRSF13C	1.9
PLIN2	1.9
ESR2	1.8
SGCG	1.8
DSCC1	1.8
C1orf162	1.8
TMEM38A	1.8
TRIM71	1.8
PLIN5	1.8
BDH1	1.8
ZMAT4	1.8
GPR157	1.8
OPLAH	1.8
PGF	1.8
GLCCI1	1.8
SHD	1.8
TSPEAR	1.7
SLFN13	1.7
RTN4RL1	1.7
FAM131C	1.7
ESCO2	1.7
PFKFB2	1.7
NOTUM	1.7
NEFL	1.7
LINC01460	1.7
RILP	1.7
FREM2	1.7
SPC25	1.7
PPIF	1.7
TMEM45B	1.7
DDN	1.7
ATP5G1	1.7
CLEC2A	1.7
SKA1	1.7

MIR1199	1.7
ASF1B	1.7
ALDH1L1	1.7
NEIL3	1.7
ECHDC3	1.7
FABP5	1.7
MYBL1	1.6
TMEM116	1.6
KCNJ8	1.6
CDCA5	1.6
MPC1	1.6
CKMT2	1.6
KIF18B	1.6
TMTC1	1.6
RNF144A	1.6
AURKB	1.6
POC1A	1.6
GOT1	1.6
SLC1A3	1.6
CA14	1.6
NT5M	1.6
PBK	1.6
TECRL	1.6
BRCA2	1.6
MLF1	1.6
NOSTRIN	1.6
OIP5	1.6
DANT2	1.6
CDK1	1.6
FITM1	1.6
PRADC1	1.6
ZNF367	1.6
POLQ	1.6
ADD2	1.6
RDH13	1.6
PARVB	1.6
UQCRHL	1.6
PPARGC1A	1.6
SLC25A19	1.6
MT-ND3	1.6
DSN1	1.5
YBX2	1.5
NAALAD2	1.5
XIRP2	1.5
ARHGAP11B	1.5
ENDOG	1.5
CDCA3	1.5

PYGM	1.5
FAM111B	1.5
PACSIN1	1.5
CRACR2B	1.5
CENPM	1.5
MRPL12	1.5
ESRRG	1.5
LINC01021	1.5
WSCD1	1.5
ESPL1	1.5
COX5B	1.5
PLK4	1.5
NLGN1	1.5
UBE2SP1	1.5
SCO2	1.5
CISD1	1.5
KIF15	1.5
GM2A	1.5
COX6C	1.5
FANCI	1.5
UBE2T	1.5
COX5A	1.5
CBWD7	1.5
MT-ND6	1.5
NDRG2	1.5
ATP5G3	1.5
MYADML2	1.5
INPP4B	1.5
ZWINT	1.5
CTSC	1.5
MRPL34	1.5
COX6B1	1.5
DPY19L2	1.5
NDUFA4	1.5
ACO2	1.5
CKS2	1.5
PNMT	1.5
C18orf54	1.5
CEP152	1.5
COX7B	1.5
HAGHL	1.5
CENPH	1.5
RAD54L	1.5
SFXN4	1.5
TYMS	1.5
RAD51AP1	1.5
HMMR	1.5

COX14	1.5
EFCAB2	1.5
MCM4	1.5
USMG5	1.5
CMC1	1.5
STARD8	1.5
TBX10	1.5
WDR62	1.5
NDUFAB1	1.5
FAM96A	1.5
SLC25A30	1.5
SLC25A5	1.5
FABP3	1.5
ABHD3	1.5
PAQR8	1.5
UQCRFS1P1	1.5
MPC2	1.5
FANCG	1.5
BUB1	1.4
EXO1	1.4
ESRRA	1.4
ACAT1	1.4
NUDT1	1.4
FAM167A	1.4
LPIN3	1.4
CYC1	1.4
PRSS45	1.4
GSTM4	1.4
TCF7	1.4
BNIP3P1	1.4
CENPK	1.4
EIF3K	1.4
LINC00982	1.4
SLC12A7	1.4
RPP25L	1.4
PRDX2	1.4
THEM6	1.4
NINJ2	1.4
OGDHL	1.4
BAIAP2L1	1.4
ETFB	1.4
MYL3	1.4
C14orf159	1.4
MRPL41	1.4
COBLL1	1.4
PCDHGC4	1.4
POLE2	1.4

COX4I1	1.4
SMC4	1.4
ATP5O	1.4
KIF20B	1.4
NDRG4	1.4
COMTD1	1.4
MRPS12	1.4
PMVK	1.4
SUV39H1	1.4
APOO	1.4
CYCS	1.4
NPM3	1.4
RAPGEF4	1.4
ADSSL1	1.4
TFAP4	1.4
NDUFS7	1.4
KIAA1524	1.4
DPY19L2P1	1.4
TXLNB	1.4
NLGN3	1.4
XRCC2	1.4
XRCC2	1.4
FASTKD1	1.4
ITGA6	1.4
NDUFB5	1.4
HSPE1	1.4
C21orf58	1.4
NDUFA3	1.4
NDUFV1	1.4
WNK2	1.4

Table S3B Genes down-regulated in CD36hi CMs, p<0.05
Fold Change is calculated as CD36hi/CD36lo CMs

Gene Symbol	Fold change
CXCL14	0.08
STMN2	0.08
PTPRZ1	0.08
CCL2	0.09
L1CAM	0.09
COL8A1	0.10
MSLN	0.11
GBP2	0.11
C3	0.11
ELF4	0.12
FXYD5	0.12
STMN3	0.13

TRPA1	0.13
PTX3	0.13
NRG1	0.13
DCLK1	0.14
CHST15	0.14
ITGA8	0.14
MYD88	0.14
FOXC1	0.14
MAMLD1	0.14
CEMIP	0.15
BMP2	0.15
MAOA	0.15
MRVI1	0.15
BMP4	0.15
CDC42EP1	0.15
MGP	0.15
CDH6	0.16
SNTB1	0.16
S100A11	0.16
CDH5	0.17
ST6GALNAC5	0.17
GNG2	0.17
ANXA1	0.17
AMIGO2	0.17
FBLN5	0.17
MFAP5	0.18
TRIM38	0.18
INMT	0.18
CHRDL2	0.18
MXRA5	0.18
OGN	0.19
COL3A1	0.19
ITGA11	0.19
KIF5C	0.19
FER1L6	0.19
FN1	0.20
MMP2	0.20
PAPPA	0.20
ALDH1L2	0.20
DSC3	0.20
OSMR	0.20
EMILIN1	0.20
PDYN	0.20
AQP3	0.21
GATA2	0.21
PROCR	0.22
EHD2	0.22

DHRS9	0.22
P4HA3	0.22
BDKRB2	0.22
PLAT	0.22
FAM19A5	0.22
TMEM59L	0.23
SLC6A4	0.23
CYP1B1	0.23
BNC2	0.23
LINC01013	0.23
ATP8A2	0.23
ST6GAL2	0.23
PELI2	0.24
TRAF5	0.24
TBX18	0.24
SGK223	0.24
LURAP1L	0.24
DCX	0.24
COTL1	0.24
KIAA1462	0.24
CBLN2	0.25
FZD7	0.25
ALDH1A2	0.25
ITGA1	0.25
ACTA2	0.25
LRRTM1	0.25
MAB21L2	0.25
HS3ST3B1	0.25
TFPI2	0.25
HAS2	0.25
SHANK2	0.26
COL6A6	0.26
NSG1	0.26
RAI2	0.26
CORO2A	0.27
RGS4	0.27
GDF6	0.27
LINC00152	0.27
NPR1	0.27
GBP4	0.28
POSTN	0.28
COL26A1	0.28
CPA4	0.28
CMKLR1	0.28
CNTNAP2	0.29
DRAM1	0.29
LGALS1	0.29

ZNF469	0.29
MAPK11	0.29
SLC9A3R1	0.29
AMOTL1	0.29
TRIL	0.29
GRM1	0.30
MYO10	0.30
GPRC5A	0.30
TENM2	0.30
EGFR	0.30
CD200	0.30
ITGA2	0.31
ITPKB	0.31
PDZD4	0.31
SEZ6L2	0.31
FAM26E	0.31
TNFRSF10D	0.31
UG0898H09	0.31
DGKG	0.31
SLC6A20	0.31
TNFRSF11B	0.31
DAAM2	0.32
SCN7A	0.32
LSAMP	0.32
GAP43	0.32
LRRN1	0.32
NEFH	0.32
DUSP6	0.33
BOC	0.33
PLXNB3	0.33
LIMA1	0.33
HCN1	0.33
ASPN	0.34
CPXM1	0.34
NRXN1	0.34
MYBPHL	0.34
C2orf82	0.34
PRUNE2	0.34
C1RL	0.34
SLC1A1	0.34
SGSM1	0.35
MYC	0.35
PRMT8	0.35
CSGALNACT1	0.35
STC2	0.35
CTGF	0.35
CDHR1	0.35

GAREM2	0.36
KDR	0.36
UNC5C	0.36
EDIL3	0.36
FIBIN	0.36
DOCK11	0.36
GRM2	0.36
IL6R	0.36
GPR158	0.37
COLEC12	0.37
FOXF2	0.37
NRIP3	0.37
ACSM3	0.37
PCDH18	0.38
PLSCR4	0.38
CAMK1D	0.38
MAMDC2	0.38
PNP	0.38
PDGFB	0.38
RGS9	0.39
NRP2	0.39
MYH6	0.39
SYNPO	0.39
ADAMTSL3	0.39
CD83	0.40
SULF2	0.40
LMO2	0.40
CNTN4	0.40
SLC1A5	0.40
NTRK2	0.40
PODXL2	0.40
CPE	0.40
KCNK6	0.41
MYOM2	0.41
ANKS1B	0.41
MYOF	0.41
CYYR1	0.41
SEMA3D	0.41
LIPG	0.42
SPRY4	0.42
RSPO4	0.42
DUOX2	0.42
HOXB5	0.42
ID4	0.42
CLSTN2	0.42
COL9A3	0.42
GRIK5	0.42

B4GALNT4	0.42
PAPSS2	0.42
PLXNC1	0.42
HHIP	0.42
TMCC3	0.42
FOXD1	0.42
ZNF385D	0.42
EPHB2	0.42
SGK1	0.42
PLAU	0.43
SEMA3F	0.43
KIAA2022	0.43
MEGF11	0.43
C9orf91	0.43
RAB11FIP4	0.43
TSPAN7	0.43
NFASC	0.43
ARPP21	0.43
GALNT6	0.43
MAPK10	0.43
OSBPL10	0.43
ZNF804A	0.43
LINC00535	0.44
EFNB2	0.44
EFS	0.44
TUBB2B	0.44
TGFB2	0.44
ZBTB7C	0.44
TPM4	0.44
GABRB2	0.45
ELK3	0.45
PIANP	0.45
PLD5	0.45
SDPR	0.45
COL5A1	0.45
DCBLD1	0.45
NKD1	0.45
HIST1H2BK	0.45
PRSS23	0.45
MFSD2A	0.45
PELI1	0.45
NYNRIN	0.45
SLC24A3	0.45
DRD2	0.46
RRBP1	0.46
SH3PXD2B	0.46
TNMD	0.46

MARVELD1	0.46
AKAP12	0.46
JAG1	0.46
MRC2	0.46
SPRY1	0.46
ACHE	0.46
FOXF1	0.46
LMOD1	0.46
SLC2A6	0.46
BST2	0.46
ARL4D	0.47
CTSV	0.47
MEIS3	0.47
ADRA1B	0.47
MN1	0.47
CALN1	0.47
MMP24	0.47
SGCD	0.47
SVEP1	0.47
OLFM2	0.47
ADGRA2	0.47
TTC39B	0.48
PDGFRA	0.48
SOX9	0.48
C2orf71	0.48
IGFBP4	0.48
KCNT2	0.48
NPAS3	0.48
MYO1D	0.48
HRH1	0.48
VSTM4	0.48
PODXL	0.48
ARHGDIB	0.49
IQGAP1	0.49
PTCHD1	0.49
RAB31	0.49
EXT1	0.49
FBN3	0.49
SORCS3	0.49
TBX2	0.49
DISP2	0.50
INHBA	0.50
POU2F2	0.50
AQP1	0.50
CAMSAP3	0.50
TMEM255A	0.50
RFTN1	0.50

FMNL3	0.50
CABLES1	0.50
PCNX2	0.50
SLC2A10	0.51
MCF2L2	0.51
SFXN3	0.51
SOX8	0.51
IFITM3	0.51
SCN9A	0.51
IL27RA	0.51
CLIP2	0.51
GFRA1	0.51
CXCR2	0.52
LASP1	0.52
THRB	0.52
GABRB1	0.52
FKBP10	0.52
NOTCH3	0.52
EPS8	0.52
SOD3	0.53
KIF3C	0.53
ST3GAL5	0.53
MICAL1	0.53
TLE3	0.54
FLRT1	0.54
RGS2	0.54
GOLGA7B	0.54
TENM4	0.54
NEK6	0.54
PPP1R14A	0.54
PXDN	0.54
STUM	0.54
NFIX	0.55
COL5A2	0.55
SPOCK1	0.55
RMDN2	0.55
SLC2A13	0.55
OLFM1	0.55
SH3BGRL3	0.55
ADAMTS7	0.55
CPNE5	0.55
MEX3B	0.55
C2CD2	0.55
TBX1	0.55
TNFRSF21	0.55
BIN1	0.55
FLRT2	0.55

GRIA3	0.56
LY6E	0.56
ADAMTS14	0.56
SCN3B	0.56
SV2C	0.56
TMEFF1	0.56
HRK	0.56
BCAR3	0.56
CLIP3	0.56
DENND2A	0.56
KIAA1024	0.56
KNDC1	0.56
PAPSS1	0.57
IGSF5	0.57
LRRC17	0.57
RERG	0.57
THSD1	0.57
CRMP1	0.57
PLS3	0.57
SLC22A17	0.57
FSTL1	0.57
IL13RA1	0.57
SORL1	0.57
CAPN5	0.57
NFKBIZ	0.57
TNFAIP3	0.58
ITGA10	0.58
SEMA4F	0.58
COL14A1	0.58
OPN3	0.58
SLC9A7P1	0.58
FEZ1	0.58
THBS1	0.58
BASP1	0.58
TENM3	0.58
SPINT2	0.59
STX1B	0.59
ITGA3	0.59
GLI2	0.59
PSD	0.59
BTBD11	0.59
L3MBTL3	0.59
LYN	0.59
PKDCC	0.59
ACTN1	0.59
PLPPR2	0.59
ST3GAL1	0.59

THBS4	0.59
MAML3	0.60
LITAF	0.60
ARPC1B	0.60
IRS1	0.60
ARNT2	0.60
GPR176	0.60
FAT3	0.60
P4HA2	0.60
ZFHX2	0.60
ARID5B	0.60
LEPR	0.60
ANOS1	0.61
FLNA	0.61
GFOD1	0.61
SYT11	0.61
APBA1	0.61
AMOT	0.61
CAP1	0.61
LRP1	0.61
CGNL1	0.62
CHRNA3	0.62
KIAA0930	0.62
KRT8P3	0.62
BTN2A3P	0.62
CHSY1	0.62
DPYSL3	0.62
SLIT3	0.62
TRPS1	0.62
CPD	0.62
FUT8	0.62
SEMA7A	0.62
DGKI	0.62
PFKFB3	0.63
TSPAN18	0.63
ADAMTS3	0.63
C4orf48	0.63
G0S2	0.63
LRRC4C	0.63
PITX2	0.63
TINAGL1	0.63
NID2	0.63
SH3BP5	0.63
FAR2	0.63
KIRREL	0.63
PRKG1	0.63
ITGB5	0.63

ACTN4	0.63
KCTD12	0.63
ASS1	0.64
GALNT10	0.64
ITPR2	0.64
MAP3K1	0.64
SLC7A8	0.64
ZSWIM5	0.64
HCG22	0.64
MAP1A	0.64
COL13A1	0.64
QSOX1	0.64
DOCK4	0.64
PTPRF	0.64
LIX1	0.64
SPRED2	0.64
ZNF423	0.65
MEGF6	0.65
GPR137B	0.65
ROBO1	0.65
ARHGAP31	0.65
AFAP1	0.66
RDH10	0.66
VASN	0.66
CACNA1B	0.66
FAT4	0.66
PTGFRN	0.66
C17orf58	0.66
SLC39A11	0.66
SEMA3A	0.66
TNFRSF1A	0.66
ARHGAP29	0.67
SH3BP1	0.67
PCDHA2	0.67
G6PD	0.67
GALNT2	0.67
CTH	0.67
GATM	0.67
JPH3	0.67
RAB11FIP1	0.67
GLIPR2	0.67
VCAM1	0.67
ITPR1	0.68
TMEM98	0.68
LAMA5	0.68
MMP15	0.68
MYCBP2	0.68

AGRN	0.68
CSPG5	0.68
LRRC1	0.68
CTSO	0.68
SEMA5A	0.68
SERPINH1	0.68
SNPH	0.69
FAM20A	0.69
SH3BGRL	0.69
AXL	0.69
CHPF	0.69
ELOVL5	0.69
FERMT1	0.69
PANX1	0.69
SUMF1	0.69
HUNK	0.69
PARVA	0.69
COL16A1	0.69
MGAT3	0.69
NUDT11	0.69
CACNB3	0.69
DDR1	0.69
DFNA5	0.70
PLD2	0.70
BAMBI	0.70
NEO1	0.70
FRAS1	0.70
PCDHB10	0.70
PTN	0.70
MANSC1	0.70
PPP1R18	0.70
ZNF503	0.70
ZNF703	0.70
C11orf24	0.70
PLOD1	0.70
TUBB6	0.70
TWSG1	0.70
MASP1	0.71
PARD3B	0.71
PLBD2	0.71
CREB3L2	0.71
MAN1C1	0.71
SLC7A2	0.71
PXDC1	0.71

Gene Symbol	r	t	pval
PPIF	0.93	8.23	9.13E-06
COX5A	0.93	7.77	1.51E-05
UQCRFS1	0.92	7.62	1.80E-05
UQCRC1	0.89	6.12	1.12E-04
CYCS	0.88	5.75	1.86E-04
CYC1	0.86	5.29	3.51E-04
COQ9	0.86	5.23	3.85E-04
NDUFA8	0.84	4.97	5.64E-04
SDHA	0.84	4.91	6.14E-04
NDUFB9	0.84	4.85	6.75E-04
NDUFV1	0.84	4.83	6.91E-04
NDUFA10	0.83	4.79	7.32E-04
NDUFS2	0.83	4.79	7.34E-04
UQCRB	0.82	4.55	1.05E-03
NDUFS6	0.78	3.88	3.06E-03
MTCH2	0.77	3.80	3.49E-03
UQCRH	0.77	3.76	3.74E-03
ATP5O	0.76	3.74	3.83E-03
COX4I1	0.76	3.74	3.86E-03
NDUFA12	0.76	3.72	3.96E-03
SDHC	0.76	3.71	4.02E-03
COX5B	0.73	3.42	0.007
CHCHD10	0.73	3.34	0.008
MSH2	0.70	3.14	0.011
DLD	0.70	3.13	0.011
NDUFS8	0.68	2.95	0.015
NDUFS1	0.66	2.79	0.019
COX7C	0.65	2.72	0.022
COX6A1	0.65	2.69	0.023
UQCR10	0.64	2.64	0.025
FXN	0.63	2.58	0.027
SDHD	0.62	2.52	0.030
COX7A2	0.61	2.46	0.034

Table S4. Pearson correlation between CD36 and genes involved in oxidative phosphorylation (GO:0006119) among CD36^{mixed}, CD36^{hi}, CD36^{lo} CMs. R=correlation coefficient, t= t statistic, pval = p-value. Genes with pval < 0.05 are shown.

**Flow Cytometry**	**Supplier**	**Cat #**	**Vol/100 µl cells**
TNNT2	Abcam	ab8295	1
CD36-APC	BD Biosciences	550956	10
APC Mouse IgM, κ isotype Control	BD Biosciences	555585	10
SIRPA1 (anti-human CD172a/b)	Biolegend	323806	2
PE mouse IgG1, κ isotype Ctrl (FC)	Biolegend	400114	2

**Immunostaining**	**Supplier**	**Cat #**	**Dilution factor**
CD36	BD Biosciences	555453	1:50
α-actinin	Abcam	ab9465	1:200
KI67	Neomarkers	RM-9106-S0	1:200
ACADVL	Abcam	ab188872	1:100
MLC2A	Synaptic systems	311011	1:50
MLC2V	Proteintech	10906-1-AP	1:100

**Western blotting**	**Supplier**	**Cat #**	**Dilution factor**
CD36	Cell Signaling	14347	1:1000
ACADVL	Abcam	ab188872	1:1000
SDHB	Abcam	ab110411	1:200

Table S5. List of antibodies used in the manuscript.
